# A toolbox for multiplexed super-resolution imaging of the *E. coli* nucleoid and membrane using novel PAINT labels

**DOI:** 10.1038/s41598-018-33052-3

**Published:** 2018-10-03

**Authors:** Christoph K. Spahn, Mathilda Glaesmann, Jonathan B. Grimm, Anthony X. Ayala, Luke D. Lavis, Mike Heilemann

**Affiliations:** 1 Institute of Physical and Theoretical Chemistry, Goethe-University Frankfurt, Max-von-Laue-Str. 7, 60438 Frankfurt, Germany; 2 Janelia Research Campus, Howard Hughes Medical Institute, 19700 Helix Drive, Ashburn, Virginia 20147 USA

## Abstract

Maintenance of the bacterial homeostasis initially emanates from interactions between proteins and the bacterial nucleoid. Investigating their spatial correlation requires high spatial resolution, especially in tiny, highly confined and crowded bacterial cells. Here, we present super-resolution microscopy using a palette of fluorescent labels that bind transiently to either the membrane or the nucleoid of fixed *E. coli* cells. The presented labels are easily applicable, versatile and allow long-term single-molecule super-resolution imaging independent of photobleaching. The different spectral properties allow for multiplexed imaging in combination with other localisation-based super-resolution imaging techniques. As examples for applications, we demonstrate correlated super-resolution imaging of the bacterial nucleoid with the position of genetic loci, of nascent DNA in correlation to the entire nucleoid, and of the nucleoid of metabolically arrested cells. We furthermore show that DNA- and membrane-targeting labels can be combined with photoactivatable fluorescent proteins and visualise the nano-scale distribution of RNA polymerase relative to the nucleoid in drug-treated *E. coli* cells.

## Introduction

Knowing how proteins and the chromosome organise in space and time is crucial to understand fundamental cellular processes. This is challenging especially in bacteria because of their small size, which limits studies with conventional optical microscopy. Super-resolution microscopy techniques can capture biomolecular organization at a much smaller scale^1,2^, but require robust labelling methods that allow high-density targeting of cellular biomolecules.

For visualisation of the bacterial nucleoid, previous works used DNA binding fluorescent probes or genetically labelled nucleoid-associated proteins to study chromosome dynamics in living cells^3,4^. Metabolic labelling facilitated super-resolution imaging of nascent DNA in fixed specimen using single-molecule localisation microscopy (SMLM)^5,6^. While covalent labelling of target structures with organic dyes or fluorescent proteins represents a convenient strategy^7^, it limits the number of probes incorporated into a sample. This affects label density and ultimately the achievable spatial resolution. An alternative technique is point accumulation for imaging in nanoscale topography (PAINT)^8,9^, in which fluorophores bind highly specific, but transiently, to the target structure. At appropriate concentrations (typically pM to nM), the dynamic binding leads to sparse detection events of single fluorophores that allow reconstructing a high-resolution image. Despite their low abundance, PAINT labels remain present in the imaging buffer in a sufficient concentration to allow for a constant exchange of bleached fluorophores, thereby enabling a high labelling density at the target structure^9^.

Initially used for the visualisation of lipid structures^9^, the basic concept of PAINT has been transferred to various novel fluorescence microscopic approaches. Examples are the transient binding of fluorescently labelled ligands^10^, target-specific peptides^11^, or short fluorescently labelled oligonucleotides^12^ to generate the single-molecule signal required for reconstruction of super-resolved images. Unfortunately, approaches that require large labels such as oligonucleotide-conjugated antibodies are difficult to apply in bacterial cells due to the cell wall, which represents an entry barrier, and the high molecular crowding in the bacterial cytosol^13^. Therefore, PAINT imaging in bacteria is limited to smaller labels that specifically bind to target structures because of their inherent affinity. As one example, the membrane of living *Caulobacter crescentus* cells was visualised using the hydrophobic dye Nile Red^8^. Furthermore, binding-activated localisation microscopy (BALM)^14^ provided the first super-resolution images of the *E. coli* nucleoid by exploiting the dynamic binding of small intercalating fluorescent dyes to double-stranded DNA (dsDNA). While the latter study represented a major step in improving the image resolution of the bacterial nucleoid, the intercalator fluorophores required imaging buffers with specific chemicals, limiting combinations with other SMLM techniques such as photoactivated localisation microscopy (PALM)^15^. Furthermore, the BALM probes are exclusively excited with blue light and hence provide limited spectral flexibility in multi-colour SMLM experiments. Recently, Hoechst-conjugated silicon-rhodamine dyes were reported to enable PAINT imaging of eukaryotic chromatin^16^, mediated by their significantly reduced affinity to dsDNA (~1000 fold) in comparison to Hoechst alone^17^. However, their applicability to bacterial organisms has not yet been shown.

A current challenge in SMLM is the combination of multiple types of probes in multi-colour approaches, especially when using different classes of labels such as organic dyes and fluorescent proteins^18^. These challenges include e.g. spectral interference, photobleaching and incompatible imaging buffers, which arise when having all labels present at a time (referred to as parallel imaging in this manuscript). These drawbacks were partially overcome in sequential labelling and/or imaging approaches, however, this requires additional efforts to either robustly relocate the same region of interest or the use of microfluidic systems^6,8^.

In this study, we present parallel multi-colour super-resolution imaging of the bacterial nucleoid and cell membrane via PAINT^8,9^. We applied labels that cover a large spectral bandwidth to target DNA and membrane, and demonstrate super-resolution imaging of multiple targets using simplified imaging workflows. We show that these probes can be used in different imaging buffers, allowing the combination of PAINT with other SMLM techniques such as *direct* stochastic optical reconstruction microscopy (*d*STORM)^19^ and PALM^15^. In contrast to metabolic labelling strategies for DNA, PAINT labelling does not require the incorporation of modified nucleoside analogues, simplifying the experimental procedure and facilitating visualisation of the bacterial nucleoid also under non-replicative conditions (e.g. during metabolic arrest^20^). However, we also show a beneficial combination of the two approaches and visualise nascent DNA in correlation to the entire nucleoid. Furthermore, the position of specific chromosomal loci can be correlated to the highly resolved nucleoid by applying the PAINT labels to bacterial strains carrying a genetic loci reporting system^21^. Finally, we demonstrate that membrane- and DNA-targeted PAINT labels can be robustly combined with PALM in a parallel imaging approach. This combination allows studying the spatial correlation of bacterial proteins to the nucleoid under various cellular conditions, as exemplarily shown for the *E. coli* RNA polymerase. Inhibition of transcription and translation led to an altered nucleoid morphology accompanied with redistribution of RNA polymerase molecules. These applications demonstrate that the presented toolbox provides nanoscale information which is valuable for the investigation of fundamental biological processes in bacteria.

## Results and Discussion

### PAINT labels facilitate super-resolution imaging of the bacterial nucleoid and membrane

**Figure 1:**
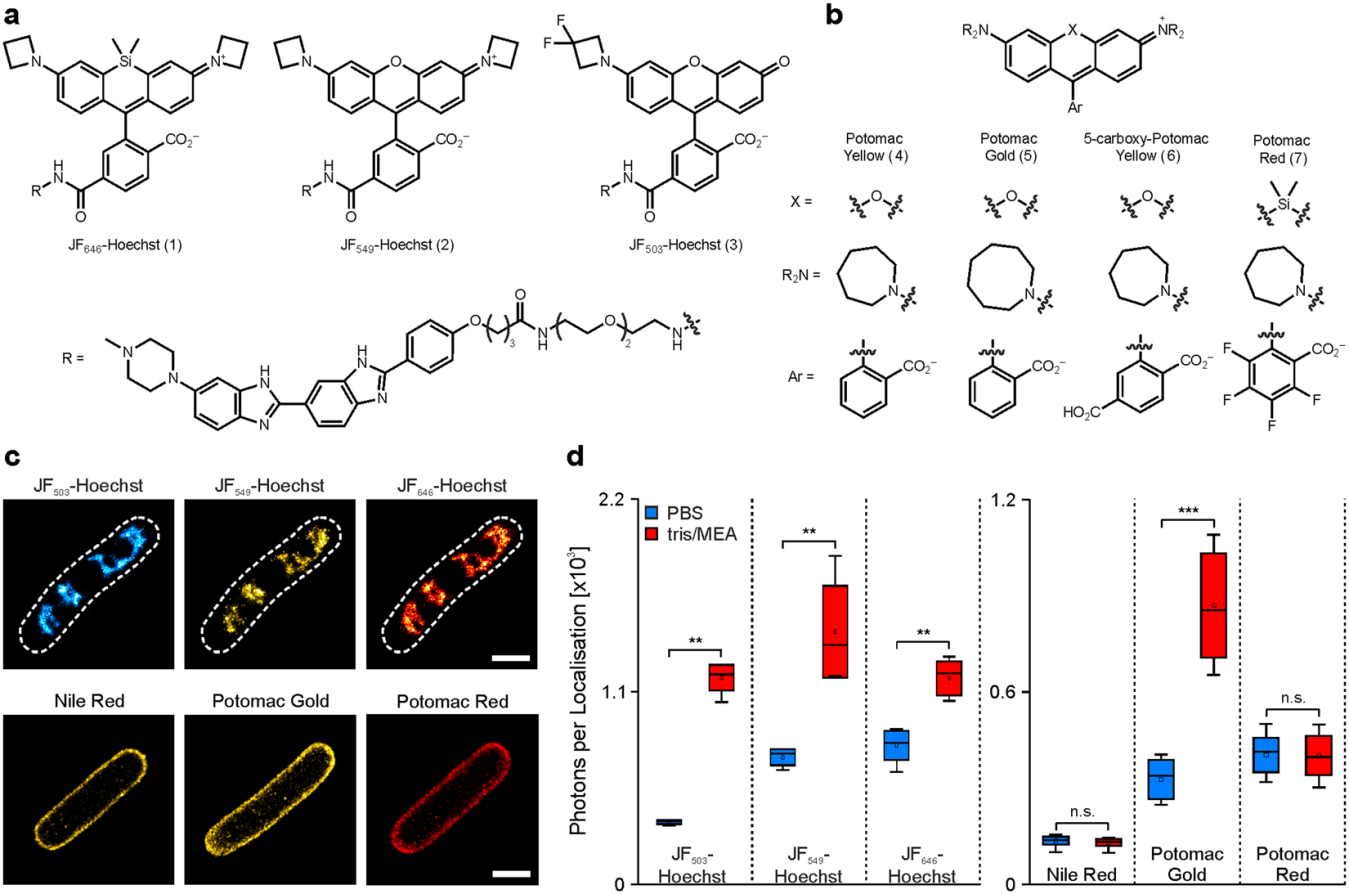
Super-resolution imaging of the bacterial nucleoid and cell membrane with novel DNA and membrane PAINT dyes in fixed *E. coli* cells, grown in LB medium. (**a,b**) Chemical structures of PAINT labels for DNA (**a**, **1**–**3**) and membranes (**b**, **4**–**7**). (**c**) The nucleoid of a fast-grown *E. coli* cell was visualised with PAINT microscopy using the Hoechst-conjugated rhodamine dyes JF_503_-Hoechst, JF_549_ -Hoechst and JF_646_-Hoechst. The dashed lines indicate the cell boundaries and were determined from PAINT images of the cell membrane (see Fig. S3). Super-resolved images of the bacterial membrane were reconstructed from PAINT measurements using the hydrophobic fluorophores Nile Red, Potomac Gold and Potomac Red (scale bar: 1 µm). (**d)** Single-molecule photon yields of the DNA-binding (left) and membrane-binding PAINT probes (right) shown in (**a**). Statistical significance was assessed using two tailed, non–parametric Mann–Whitney test: n.s. P > 0.05; **P < 0.01; ***P < 0.001. Rectangles represent mean values and boxes the respective standard deviations. The median is indicated by horizontal lines, while whiskers indicate the minimal and maximal values.

We introduce known and novel rhodamine-based probes^22^ that facilitate PAINT imaging of the *E. coli* nucleoid and membrane (Fig. 1). We expand previous work establishing two separate classes of PAINT probes^16^ (Fig. S1). For DNA binding, we originally coupled the Si-rhodamine dye Janelia Fluor 646 (JF_646_) to Hoechst 33342, which binds the minor groove at AT-rich sequences^16,23,24^, to prepare JF_646_-Hoechst (1). We expanded this concept to other Janelia Fluor dyes with different spectral properties (Figs S1 and S2), yielding JF_549_-Hoechst (2) and JF_503_-Hoechst (3, Fig. 1a). For membrane targeting, we synthesised derivatives of the previously described azepanyl-rhodamine dye ‘Potomac Yellow’ (4), which we serendipitously discovered to have inherent affinity for cellular membranes^16^. This structure was modified to increase hydrophobicity (‘Potomac Gold’, 5), introduce a negative charge (5-carboxy-Potomac Yellow, 6), or red-shift spectra (‘Potomac Red’, 7, Figs 1b and S2). These probes allowed us to visualise the nano-scale structure of the *E. coli* nucleoid and cell envelope with localisation precisions in the range of 10–20 nm (Figs 1, S3, S4 and Table S1), providing significantly enhanced resolution compared to conventional fluorescence microscopy techniques such as confocal laser scanning microscopy (Fig. S5). A sequential multi-colour SMLM approach^6^ enabled repeated imaging of nucleoids within the very same cell using different labels and buffer conditions. We could show that JF_503_-Hoechst, JF_549_-Hoechst, and JF_646_-Hoechst are well suited to resolve the nano-scale structure of the nucleoid in fast-grown *E. coli* cells (Fig. 1c). In addition, we found that the novel dyes perform well both in neutral (PBS, pH 7.4) and reducing (here 100 mM tris, 100 mM MEA, pH 8.0) buffer systems (Fig. S3). This finding is important for multiplexed imaging in combination with other SMLM techniques such as *d*STORM and PALM. PBS may be preferred when using photomodulatable fluorescent proteins, since reducing buffer systems might affect photo-activation/-conversion during PALM experiments^18^. In contrast, most organic dyes in *d*STORM imaging require reducing buffer systems for efficient transfer into a long-living dark state. Additionally, we found that the Hoechst conjugates show significantly increased brightness in tris/MEA buffer compared to PBS. This increase is not contributed to improved photoswitching behaviour as seen in *d*STORM experiments, but rather to the altered nano-environment of the Si-rhodamine dyes. Although spontaneous photoswitching was reported for spirolactones in eukaryotes^25^, we could not observe such behaviour for covalently incorporated JF_646_. Hereby, the photobleaching dynamics observed in neutral (PBS) or reducing buffer (tris/MEA) were similar, indicating that no transfer to a long-living dark state was induced (Fig. S6). The stable signal observed for the Hoechst conjugates is therefore most probably contributed to the transient binding, supported by the µM affinity to dsDNA reported in previous studies^16,17^ We further analysed the binding times, which showed similar distributions for the membrane- and DNA targeting probes and the different buffers (Fig. S7). The higher number of photons collected per single fluorophore leads to an improved experimental localisation precision (Fig. 1d, Table S2) with Hoechst-JF_503_ showing the strongest change from about 18.0 ± 1.0 nm to 11.9 ± 0.3. nm. An increase in brightness in tris/MEA buffer was also observed for the membrane dyes Potomac Yellow, 5-carboxy-Potomac Yellow, and Potomac Gold (Figs 1d and S3). While all membrane PAINT dyes can be used to visualise the cell envelop in 2D PAINT measurements, the increased brightness of e.g. Potomac Gold in comparison to Nile Red is beneficial for 3D SMLM measurements (Fig. S8).

For the Hoechst conjugates, optimal imaging conditions were found for concentrations of 400 to 800 pM (Fig. S9). However, these values depend on parameters such as cell fixation and permeabilisation strength or the applied growth conditions. We therefore recommend a titration prior to an experiment, in order to achieve a sufficient label density and at the same time prevent overlapping single-molecule signals^26^. At optimal dye concentrations, the permanent label exchange ensures a constant emitter density that allows for long image acquisitions. In addition, PAINT imaging is not affected by irreversible photobleaching as observed for covalently bound organic fluorophores (“stationary labels”) (Figs S6 and S10). The binding times of the PAINT labels (~20–50 ms, Fig. S7) allow for image acquisition rates commonly used for conventional *d*STORM measurements or peptide-based PAINT (e.g. lifeact, 23 ms halflife time)^11^. Other techniques such as PALM and DNA-PAINT often employ longer camera integration times (~100–200 ms)^12,27^.

### Comparison of the nucleoid structure observed in live-cell and PAINT imaging

**Figure 2:**
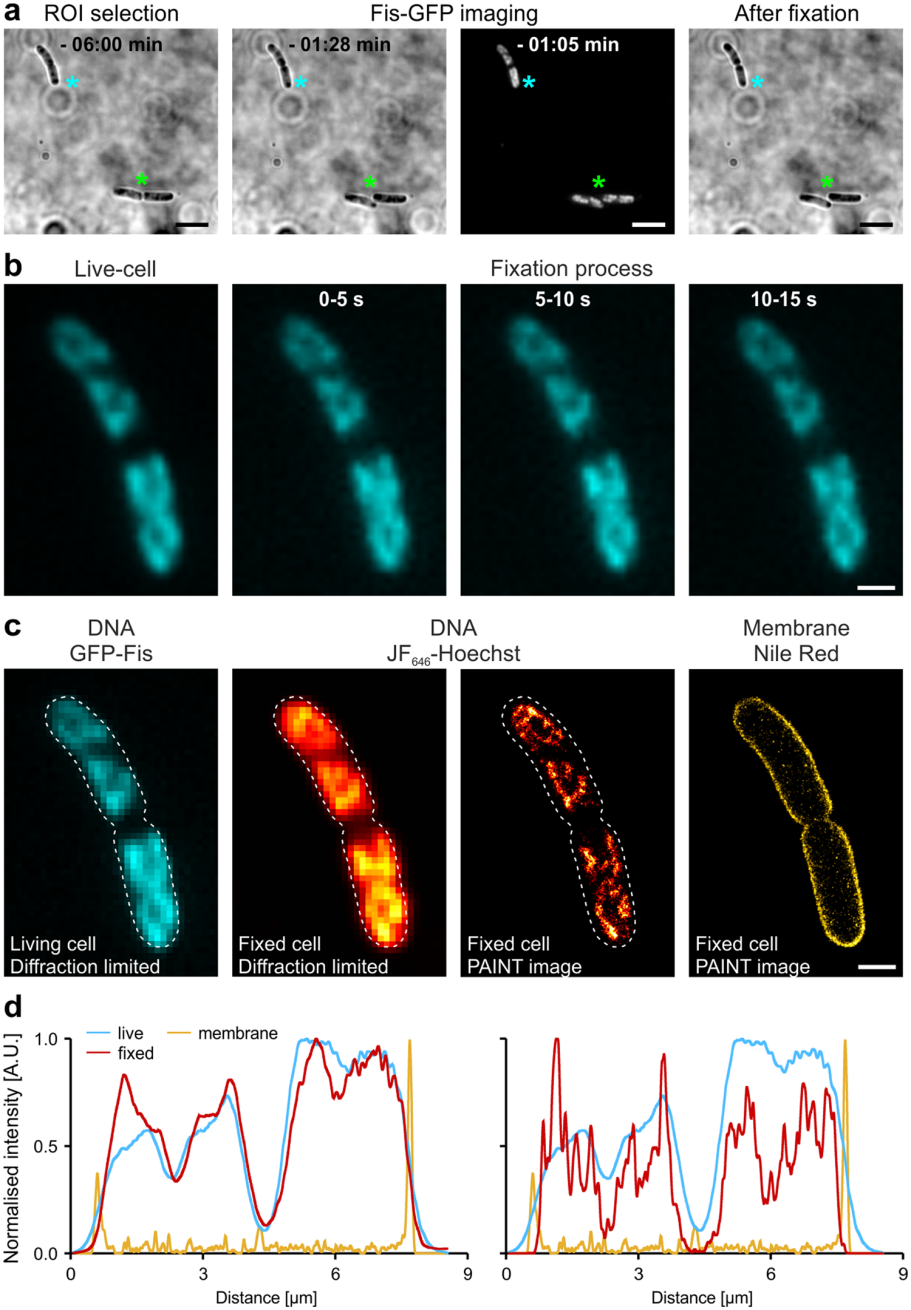
Influence of formaldehyde fixation on nucleoid morphology and distribution. (**a**) Living MG1655 wildtype cells grown in LB medium at 32 °C and expressing GFP-Fis immobilised on a chitosan-coated surface. Cells maintained cell growth and division (green asterisk). (**b**) Cells were fixed on-the-slide while concomitantly recording the GFP-Fis signal (cyan hot). Time stamps indicate the time after formaldehyde addition, each image represents an average of 5 imaging frames. The bacterium shown corresponds to the bacterium marked by cyan asterisk in (**a**). (**c**) Comparison of GFP-Fis signal recorded prior to fixation with the respective PAINT image acquired using JF_646_-Hoechst after 30 min FA fixation. A diffraction limited image of the PAINT channel was reconstructed by calculating the standard deviation image from 5,000 imaging frames. White dashed lines indicate the outlines determined from the membrane PAINT image, which was acquired in parallel imaging mode using Nile Red (scale bars: 3 µm in a and 1 µm in b and c). (**d**) Normalised intensity profiles of the cell shown in (**c**). Profiles were determined along the bacterial long axis for the GFP-Fis signal recorded in the living cell (cyan lines, both plots), the reconstructed diffraction-limited PAINT signal of DNA (red line, left plot), the super-resolved PAINT signal of DNA from the fixed cell (red line, right plot) and the PAINT signal of the membrane (yellow lines, both plots).

A highly structured morphology of the *E. coli* nucleoid during fast growth was reported by several studies^6,28,29^. Application of PAINT labels in this study and *d*STORM imaging of click-labelled DNA revealed that DNA is highly compacted and forms intriguing nucleoid structures. We aimed to test whether these structures natively occur in living cells and are conserved during chemical fixation. Therefore, we expressed a GFP-Fis fusion protein from an inducible plasmid in the MG1655 WT strain. GFP-Fis uniformly decorates the bacterial nucleoid and allows indirect visualization of DNA structure in living cells using conventional fluorescence microscopy^28^. Expression of GFP-Fis was induced in a fast-growing culture and living cells were subsequently immobilised on chitosan-treated surfaces^30^ (Fig. 2). After recording brightfield and GFP-Fis widefield images (Fig. 2a), cells were chemically fixed using 2% formaldehyde.

Prior to fixation, immobilised bacteria continued to grow and divide, excluding major inhibitory effects caused by cell immobilization (Fig. 2a, green asterisk, Fig. S11). Direct acquisition of the GFP-Fis channel upon addition of the fixation solution did not reveal changes in cell and nucleoid morphology (Fig. 2b). Despite the temperature shift during immobilization and live-cell image acquisition (32 °C to room temperature), the nucleoid revealed the highly structured morphology reported in previous studies^6,28,29^. This is consistent with published results^28^ showing that the overall geometry of the nucleoid persists over a few-minute timescale. After 30 minutes of fixation, the sample was removed from the microscope, cells were permeabilised and JF_646_-Hoechst/NR containing imaging buffer was added to record super-resolved PAINT images of the bacterial nucleoid and membrane (Fig. 2c). As expected, highly condensed DNA filaments shape the two nucleoids, which were already separated at this cell cycle stage. To exclude that chemical fixation or the binding of Hoechst induce DNA condensation during and after the fixation process, we reconstructed a diffraction-limited image from the PAINT image sequence. This allows a direct comparison with the widefield image captured in the living cell (Fig. 2c), showing very high similarity in nucleoid structure and distribution (Fig. 2d). This provides direct evidence that the super-resolved snapshots in fixed cells visualised by both *d*STORM^5,6^ and PAINT, represent natively occurring structures of the bacterial nucleoid. Such controls are important, since chemical fixation using crosslinking reagents is often considered to cause imaging artefacts and may affect nucleoid morphology and distribution. At the same time, chemical fixation is largely accepted in biochemical experiments such as chromosome conformation capture^31^.

### Spectrally separated PAINT labels allow for multiplexed imaging of genetic loci, the nucleoid and the membrane

**Figure 3:**
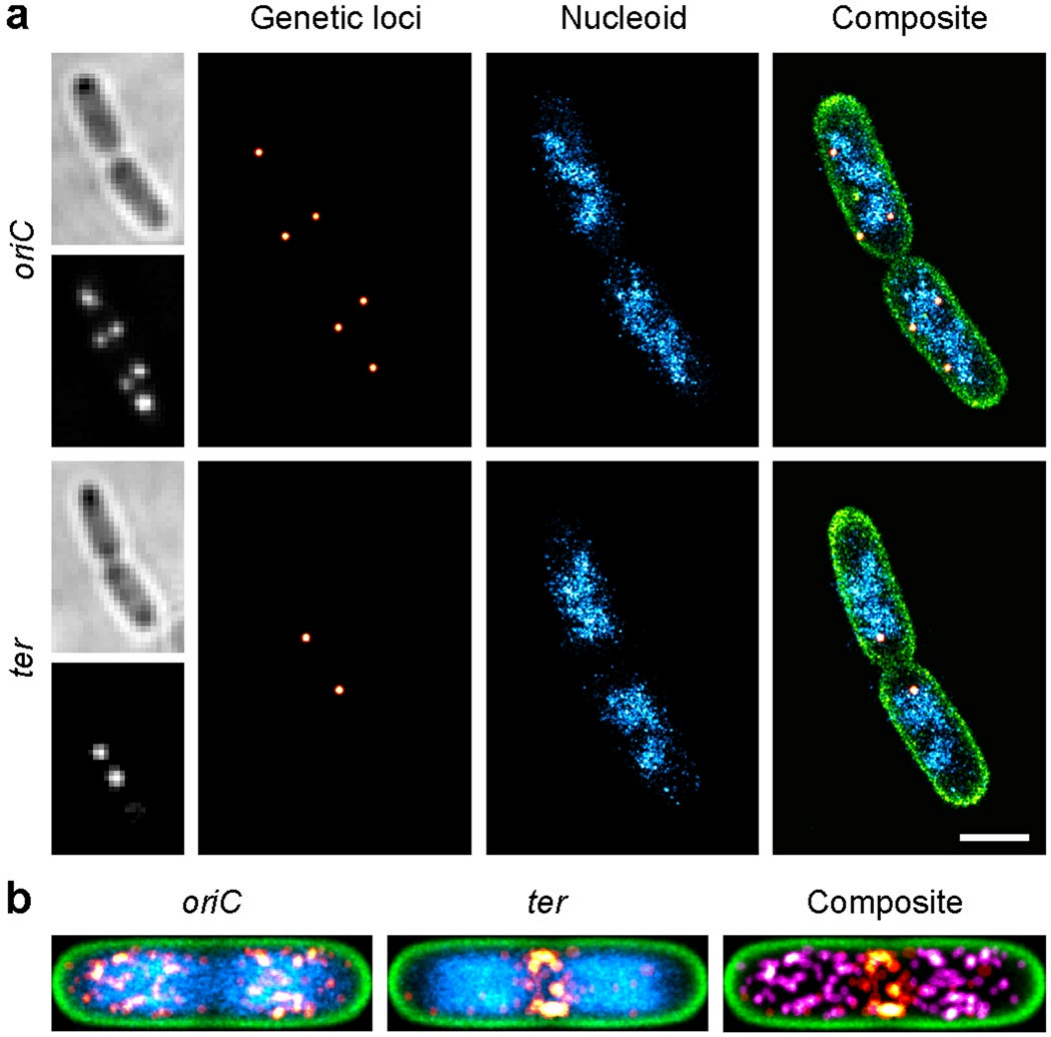
Parallel multi-colour SMLM of genetic loci, the nucleoid and the membrane in fixed *E. coli* cells, grown in supplemented M9 minimal medium. (**a**) The origin of replication (top panel) or the terminal region (bottom panel) of the bacterial chromosome were labelled with GFP using a *parS*-ParB genetic loci tagging system (red hot). The membrane (green) was stained with Nile Red, while the nucleoid (cyan) was stained with JF_646_-Hoechst (scale bar: 1 μm). (**b**) Average localisation maps of the origin of replication (left panel) and terminal region (mid panel). The composite (right panel) shows the exclusion of *oriC* (magenta) and *ter* regions (red hot).

Because of the well-separated absorption and emission spectra (Fig. S2), some membrane and DNA binding probes allow for parallel multi-colour SMLM. This simplifies the experimental procedure, as there is no need for buffer exchange and cumbersome retrieval of previously imaged cells. We performed parallel three-colour imaging of the cell envelope and nucleoid in *E. coli* MG1655 cells carrying a *parS*-ParB genetic loci tagging system^21^. The *parS* sequence in the strains used was located either near the origin of replication or the terminal region of the *E. coli* chromosome. GFP-ParB fusion proteins specifically bind to *parS* sequences and report the position of the chromosomal loci. These positions were localised and correlated to the entire nucleoid as well as the cell boundary (Methods section). Culturing *E. coli* cells in supplemented minimal medium (culture mass doubling times t_d_ = 46.6 ± 0.9 and 48.2 ± 1.2 min for Ori-1 and Ter-5, respectively) led to an apparently less condensed and less complex nucleoid structure (Fig. 3) in comparison to cells grown in rich medium (Figs 1 and 2, t_d_ = 27 min)^6,28^. At this intermediate growth rate, we observed 2–6 origins of replication per cell, while at maximum two terminal regions were found (Figs 3a and S12). In order to investigate the relative positioning of Ori and Ter macrodomains, we generated normalised localisation maps by averaging the super-resolved images of several cells (Fig. 3b, n = 44 and 45 cells for *ori*- and *ter*-tagged cells). The variation in the spatial position of the Ori macrodomain (Fig. 3b, left panel) hereby arises from the varying number of replication cycles per cell and the normalization and averaging of cells of different lengths (Fig. S12). In contrast, the Ter macrodomain remained positioned near mid-cell (Fig. 3b, mid panel), with *ter* loci being spatially separated from *oriC* (Fig. 3b, right panel).

### Multiplexed PAINT and dSTORM imaging visualises nascent DNA in correlation to the entire nucleoid

**Figure 4:**
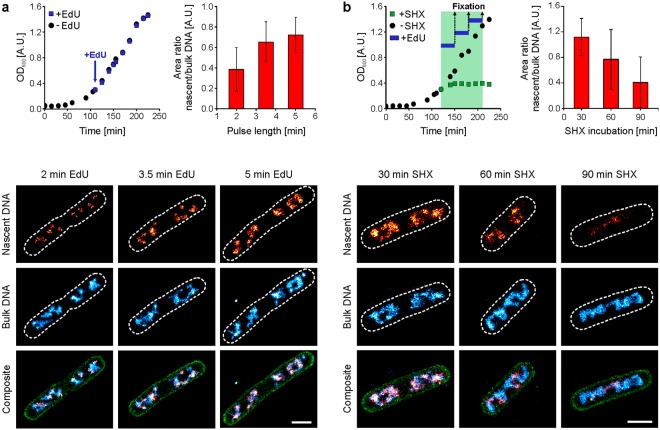
Pulse labelling of nascent DNA in *E. coli* cells with EdU. (**a**) Pulse labelling experiment in a fast-growing *E. coli* batch culture (LB medium, 32 °C). Growth curves of cultures with and without EdU are shown in the upper left panel. Culture aliquots were fixed after 2, 3.5 and 5 min and labelled with Alexa Fluor 647 via click-chemistry. Parallel multi-colour imaging was performed as described in the Methods section. Nucleoids were probed using JF_503_-Hoechst and membranes using Nile Red. Single localisations in the DNA channels were removed using a density-based cluster analysis algorithm DBSCAN^35^ (see Methods section and Fig. S13). Increasing EdU incubation time leads to an increase of nucleoid coverage (ratio of the area populated by pulse-labelled DNA and the entire nucleoid) (upper right panel). The lower panel shows representative cells for the indicated EdU pulse durations, labelled for nascent DNA (red hot), the entire nucleoid (cyan hot) and the membrane (green). Outlines were determined in the membrane PAINT image and are shown by dashed lines in the DNA *d*STORM and PAINT images. (**b**) Pulse labelling during metabolic arrest. MG1655 cells were grown in LB medium at 32 °C. Growth curves of cultures with and without SHX are shown in the upper left panel. The treated culture was split into 3 cultures and EdU was added for the last 30 min of SHX treatment, finally resulting in cells arrested for 30 min, 60 min and 90 min. Parallel multi-colour imaging was performed as described in the Methods section. The nucleoid coverage of nascent DNA decreases with the time of SHX treatment (upper right panel). The lower panel shows representative cells for the different time points during metabolic arrest, the colour-coding is kept identical to (**a**). Bars represent mean values and error bars the respecting standard deviation (scale bars: 1 µm).

Since transient binding of Hoechst conjugates is also observed in reducing buffer systems, PAINT imaging of the nucleoid can be combined with conventional *d*STORM imaging (Fig. 4). This enables imaging of the bacterial DNA with both a PAINT label and a photoswitchable fluorophore covalently bound to DNA. We utilised a common metabolic labelling approach to incorporate the thymidine analogue 5-ethynyl-2′-deoxyuridine (EdU) into nascent DNA of replicating bacteria. This metabolic labelling approach was previously applied to mammalian and bacterial cells to study the spatio-temporal organization of DNA replication using conventional fluorescence microscopy^32,33^ and SMLM^6,34^.

We have designed two experiments in which EdU was added to the culture for defined time intervals either in normally growing cultures (Fig. 4a) or during stringent response^20^ (Fig. 4b).

We used short incubation times of 2–5 minutes to label a variable fraction of newly replicated chromosomal DNA in *E. coli* cells with EdU and conjugated the organic dye Alexa Fluor 647 to the modified nucleobases using a “click chemistry” reaction^33^. This enabled us to record dual-colour SMLM images and to correlate the spatial distribution of Alexa Fluor 647-labelled nascent DNA to the entire bacterial nucleoid visualised with the PAINT dye JF_503_-Hoechst (Fig. 4a). The addition of EdU did not affect cell growth (Fig. 4a, upper left panel) and labelling controls revealed negligible contribution of unspecific binding, which was removed using a density-based cluster analysis (DBSCAN^35^) (Fig. S13).

For 2 min EdU exposure, we found foci of nascent DNA with diameters of 176 ± 7 nm (SEM, n = 287 clusters) (Fig. 4a, lower panel). This diameter is in good agreement with genetic loci mobility found in living cells^21^, which were reported to diffuse with 0.0001 to 0.0004 µm²/s. These values translate to a circular area with a diameter of 124–248 nm being covered within the time of 2 minutes. As expected, proceeding replication results in larger areas populated by nascent DNA (Fig. 4a, lower panel). Since the entire nucleoid can be visualised using the DNA-targeted PAINT labels (here: JF_503_-Hoechst), the relative nucleoid area covered by nascent DNA can be determined as the area ratio of nascent and bulk DNA area (Fig. 4a, upper right panel, Fig. S14). Also here, low-density regions of Alexa Fluor 647 and JF_503_-Hoechst signals were removed (Fig. S13). The analysis of 30–49 cells per condition revealed that increasing EdU incubation time results in successive increase of the nascent/bulk DNA area ratio. The opposite trend could be observed during metabolic arrest using the serine homologue serine hydroxamate (Fig. 4b). SHX treatment stalls translating ribosomes, which leads to inhibition of transcription by triggering the stringent response and ultimately inhibits replication initiation^20^. We observed that culture growth was immediately affected by SHX, leading to a constant optical density after ~30 min (Fig. 4b, upper left panel). To visualise the effect of amino acid starvation on chromosome replication, EdU was added to the SHX-containing growth medium 30 minutes prior to fixation. Since *E. coli* cells exhibit multifork replication under the applied growth conditions^20^ (LB medium, 32 °C), the entire nucleoid should be labelled at least once within the 30 min period of EdU exposure. Indeed, within the first 30 minutes of starvation, replication appears not to be affected, as EdU is incorporated to label the entire *E. coli* nucleoid (Fig. 4b, lower panel). However, if EdU is added for a 30 min period at the end of the SHX treatment (60–90 min), just a small part of the nucleoid is labelled, while the whole chromosome can still be visualised using JF_503_-Hoechst (Fig. 4b, lower panel). Analysis of nascent and bulk DNA of 21–53 cells per time point revealed a decreasing area ratio with SHX incubation time (Fig. 4b, upper right panel), which is consistent with termination of active replication and concomitant inhibition of new rounds of replication.

### Combination of PAINT and PALM for parallel 3-colour imaging

**Figure 5:**
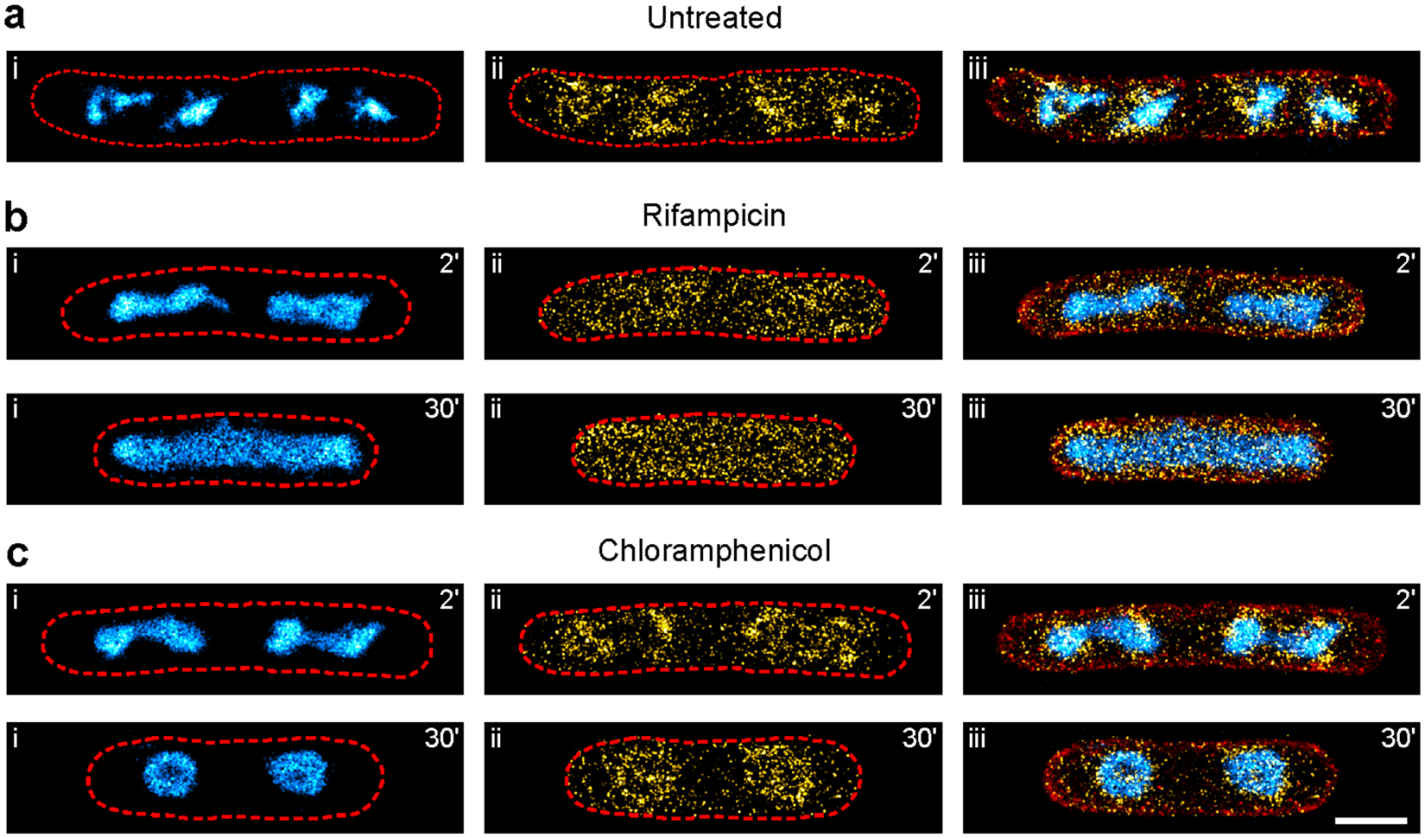
Combination of PALM and PAINT for parallel 3-colour SMLM imaging. Strain KF26 was grown in LB medium at 32 °C, chemically fixed and imaged (see Methods section). Shown are the nucleoid (i, cyan hot), the RNA polymerase β′ subunit (ii, PAmCherry1 fusion, yellow hot) and the overlay (iii) including the membrane PAINT image (red). (**a**) Representative 3-colour image of untreated KF26 cells. Cell outlines (red dashed lines) were determined using the Potomac Red membrane PAINT image. (**b**) Representative images of KF26 cells treated with 100 µg/ml rifampicin for the indicated duration. (**c**) Representative images of KF26 cells treated with 50 µg/ml chloramphenicol for the indicated duration (scale bar: 1 µm).

A sequential imaging workflow combining PALM imaging of photomodulatable fluorescent protein fusions with *d*STORM imaging of nascent DNA was reported recently^6^. Sequential imaging was necessary since the buffer system required for photoswitching of the organic dye strongly affected the photoactivation of the fluorescent protein photoactivatable mCherry1 (PAmCherry1), but this approach requires robust recovery of sample positions. Since the Hoechst conjugates do not require specific imaging buffers, PAINT imaging of the nucleoid should be compatible with PALM, significantly simplifying both sample preparation and the imaging procedure. To validate that the presence of DNA- and membrane-targeted PAINT labels in the imaging buffer does not affect PALM imaging, we overexpressed his-tagged PAmCherry1 from an inducible plasmid (Fig. S15) and quantified the PAmCherry1 signal using both the sequential and parallel imaging workflow. The results provided by both workflows were in very good agreement both qualitatively (3-colour SMLM images) and quantitatively (PAmCherry1 photon budget and number of molecules per µm² cell area) for three bandpass emission filters commonly used for PAmCherry1 detection. The photon budget per PAmCherry1 molecule hereby strongly correlated with the fraction of the emission window covered by the respective bandpass filters (Fig. S15). The identical photon budgets for PAmCherry1 in parallel and sequential imaging mode exclude significant crosstalk from the DNA- and membrane-targeted PAINT probes. This is supported by the spatial separation of cytosolic PAmCherry1, the membrane and the nucleoid as it is observed in the multi-colour images (Fig. S16). To evaluate potential crosstalk caused by other PAINT probes, we also recorded emission spectra and overlaid filter data (Fig. S17, Table S3). After validating its robustness, we used the parallel imaging approach to visualise the RNA polymerase β′ subunit fused to PAmCherry1 (now only referred to as RNAP) in correlation to the nucleoid and cell boundaries (Fig. 5). We chose this fusion protein since (i) we can compare the images to previous results obtained using the sequential imaging approach^6^ and (ii) the influence of the transcription- and translation-halting drugs rifampicin and chloramphenicol on RNAP and nucleoid distribution are well established (nicely reviewed by Jin *et al*.^36^). Using our parallel imaging approach, we observed that RNAP molecules largely organise around the *E. coli* nucleoid in untreated, exponentially growing cells, as reported by several studies^6,29,37^ (Figs 5a and S18). Addition of rifampicin inhibits transcription initiation, abolishing active transcription within 5–10 minutes of treatment^38^. Within the first two minutes of treatment, the nucleoid strongly condensed along the bacterial short axis, while RNAP molecules were distributed homogenously throughout the cytosol. A fraction of RNAP molecules resided in proximity to the nucleoid, presumably transcribing larger genes. Longer rifampicin treatment led to a uniform RNAP distribution, concomitant with expansion of the nucleoid which populated almost the entire cytosol by the time of 30 min exposure (Figs 5b, S18 and S19).

Inhibition of translation elongation using chloramphenicol initially led to a similar nucleoid morphology as observed for rifampicin treated cells, showing nucleoid condensation along the short axis. However, and in contrast to rifampicin treatment, prominent clusters of RNAP molecules were located around the nucleoid. At 30 min chloramphenicol treatment, the nucleoid morphology changed into a toroidal structure, maintaining RNAP localisation around the nucleoid (Figs 5c, S18 and S19). This morphological change was attributed to condensing forces exerted by actively transcribing RNAP molecules^37^. Note that the effects of both rifampicin and chloramphenicol treatment were independent of the cell cycle stage, as changes in nucleoid and RNAP distributions were similar for cells of all sizes (Fig. S19). Together, these results show that our parallel imaging approach provides valuable information about the nanoscale distribution of bacterial proteins in correlation to the nucleoid. PAINT labels hereby do not require any timing, as e.g. EdU, and allow for straight-forward parallel 3-colour SMLM imaging of drug-treated cells.

## Conclusion

We introduce fluorophores to visualise the bacterial chromosome and membrane using super-resolution imaging. The DNA- and membrane-targeted PAINT labels are available with different spectral properties and are thus ideally suited for multiplexed SMLM imaging. Being largely unaffected by the tested imaging buffers, these fluorophores can be combined with PALM and *d*STORM for parallel multi-colour imaging without the need for buffer exchange or sample removal. The latter combination can be used to investigate chromosome replication and segregation. Co-visualisation of nucleoid associated proteins with the nucleoid will provide insights into DNA compaction or the organisation of distinct chromosomal domains^21,39^. Furthermore, signalling pathways and their association to the membrane e.g. via membrane-bound proteins, or to the nucleoid via transcription factors could be investigated. The recently reported primed conversion, an alternative way for photoactivation in PALM, can be implemented into the parallel imaging workflow presented in this study, facilitating to visualise the nanoscale organisation of two proteins of interest in correlation to the nucleoid and membrane^40^. We are convinced that the presented labels and their application in multi-colour super-resolution microscopy will greatly contribute to the understanding of chromosome-related processes in *E. coli* and also other bacterial species.

## Methods

### Cell culture and sample preparation

Experiments were performed using the wildtype *E. coli* strain MG1655 (CGSG #6300), unless stated otherwise. Genetic loci tagging experiments were performed in MG1655 strains having a *parS* sequence inserted either near the origin of replication or the terminal region of the chromosome (a generous gift from Olivier Espeli). In these strains, a ParB-GFP fusion protein was expressed from a pALA2705 plasmid^21^. For live-cell fixation experiments, chemically competent MG1655 wildtype cells were transformed with the plasmid pZE12-GFP-*fis*^28^. Expression of His-PAmCherry1 was performed in MG1655 cells carrying the arabinose-inducible plasmid pBAD/HisB-PAmCherry1^27^ (gift from Vladislav Verkhusha (Addgene plasmid # 31931). Ampicillin (Sigma Aldrich, 100 µg/ml) was added for selection and plasmid maintenance. Strain KF26^41^ was used for 3-colour SMLM imaging of RNAP, the nucleoid and the membrane.

Bacterial cultures were inoculated from a single colony picked from an LB agar plate struck out from a glycerol stock. After incubation over night at 32 °C and shaking cultures with 230 rpm, cultures were inoculated to an OD_600_ of ~0.05–0.08 and grown to mid-exponential phase. OD_600_ measurements were carried out using a Pearl NanoPhotometer (IMPLEN) to monitor culture viability and determine culture mass-doubling times. M9 minimal medium supplemented with 0.4% glucose (Sigma Aldrich), 1 µg/ml thiamine (Sigma Aldrich) and 0.1% (w/v) casamino acids (Sigma Aldrich) was used for genetic loci tagging experiments. All other cultures were grown in LB medium. Cells were fixed for 10 min at room temperature using a mixture of 2% methanol-free formaldehyde (Thermo Fisher Scientific) and 0.05% glutaraldehyde (Electron Microscopy Sciences) in 33 mM sodium phosphate buffer (pH 7.5). For EdU labelling, 10 µM EdU (Baseclick) was added to the culture 2, 3.5, 5 (pulse labelling) or 30 min (SHX treatment) prior to fixation. For the latter experiment, SHX (Sigma Aldrich, 1 mg/ml final concentration) was added to the growth medium for 30–90 min to inhibit replication initiation^20^. After fixation, excess fixation reagents were quenched for 2 min using 0.2% (w/v) sodium borohydrate (Sigma Aldrich) in phosphate buffered saline (PBS, Sigma Aldrich). Bacteria were washed thrice with PBS and subsequently immobilised for 30 min on cleaned and poly-L-lysine (Sigma Aldrich) coated 8-well-chamberslides (Sarstedt). After two additional washing steps, immobilised cells were permeabilised for 60 min using 0.5% TritionX-100 (Sigma Aldrich) in PBS and washed twice with PBS. The DNA of the EdU treated bacteria was labelled with Alexa Fluor 647 azide (Thermo Fisher Scientific) via click chemistry as described previously^6^. For PALM experiments, cells were fixed for 30 min at room temperature using only 2% formaldehyde in the buffer described above. Excess formaldehyde was quenched for 20 min using 50 mM ammonium chloride (Sigma) in PBS. 60 and 80 nm gold beads (Nanopartz) were added to all samples and later used as fiducial markers.

### Live-cell fixation experiment

Overnight cultures of MG1655 cells carrying pZE12-GFP-*fis* were diluted 1:500 into LB medium containing 100 µg/ml ampicillin and 1 mM Isopropyl β-D-1-thiogalactopyranoside and grown at 32 °C and 230 rpm. At an OD_600_ of ~0.25, cells were concentrated 5-fold by centrifugation (2 min at 2,000 g) and immobilised for 3 min on 8-well-chamberslides which were freshly coated with 0.07% chitosan (Alfa Aesar). A temperature of 32 °C was maintained during this procedure. The chamber was washed thrice with 32 °C warm LB medium and cells were immediately transferred to the microscope. Multiple regions of interest (ROIs) were selected and positions were saved for relocation during the sequential imaging approach^6^. GFP-Fis images were recorded at low 488 nm laser densities using highly inclined and laminated sheet (HILO) illumination to reduce LB autofluorescence. After recording all ROIs, fixation solution (final concentration: 2% FA, 33 mM sodium phosphate buffer) was added while concomitantly recording the GFP-Fis signal. After 30 min fixation on the microscope, cells were permeabilised for 45 min using 0.5% Triton-X100 in PBS and washed twice with PBS. 400 pM Nile Red and JF_646_-Hoechst in tris/MEA buffer was added and PAINT imaging was performed as described below.

### Expression of cytosolic his-tagged PAmCherry1

MG1655 cells containing the arabinose-inducible plasmid pBAD/HisB-PAmCherry1 were grown in LB medium at 32 °C. At OD_600_ = 0.1, 0.01% L-arabinose (Sigma) was added to the culture for 75 min. Cells were fixed and prepared as described above (section Cell culture and sample preparation) and transferred to the microscope for both sequential and parallel multi-colour imaging. 400 pM Potomac Red and JF_503_-Hoechst were used for PAINT imaging of the membrane and nucleoid.

### Drug treatment

KF26 cells were diluted 1:500 from the ON culture into fresh LB medium lacking antibiotics and grown at 32 °C and 230 rpm. 100 mg/ml rifampicin (Sigma, catalogue number R3501, LOT number SLBP9440V) or 50 mg/ml chloramphenicol (Sigma, catalogue number C0378, LOT number SLBN6556V) stock solutions were prepared directly before use in DMSO or ethanol, respectively. At mid-exponential phase antibiotics were added (1:1000 dilution) and samples were fixed after 2 and 30 min treatment as described above. After permeabilisation, 400 pM Potomac Red and JF_503_-Hoechst in 150 mM tris pH 8.0 were added for parallel 3-colour imaging (see below).

### Determination of PAmCherry1 emission spectrum

10 µM PAmCherry1 in PBS was photoactivated using 405 nm illumination. The emission spectrum was recorded using a Cary Eclipse fluorescence spectrometer (Agilent Technologies, USA), exciting activated PAmCherry1 molecules with light of 518 nm wavelength.

### Confocal microscopy

CLSM imaging was performed on a commercial microscope (LSM710, Zeiss, Germany) equipped with a Plan-Apo 63x oil objective (1.4 NA). 100 nM Nile Red and JF_646_-Hoechst were added to fixed and permeabilised MG1655 wildtype cells. Membrane and nucleoid image channels were recorded sequentially using 543 nm and 633 nm excitation lasers and suitable filter sets. The pixel size was set to 65 nm.

### SMLM microscopy

Super-resolution imaging of all samples was performed on a home-built microscope as described previously^6^. In brief, a Coherent Innova 70 C Spectrum laser is coupled into a 100xApo TIRF oil objective mounted on a Nikon Eclipse Ti microscope body. A perfect focus system (Ti-PFS; Nikon) maintains the z-position, while the x-y-position is adjusted with a motorised stage. Excitation laser lines are selected using an acousto-optic tuneable filter (AOTF; AA Opto Electronic). By manual adjustment of a TIRF mirror, the illumination mode can be changed from widefield- over HILO- to TIRF-illumination. Excitation and fluorescence emission light is separated using a dichroic mirror and fluorescence emission is filtered using appropriate emission filters (Table S2) before it is detected on an Andor Ixon Ultra EMCCD chip (DU-897U-CS0-#BV; Andor). A customised cylindrical lens (RCX-39.0.38.0-5000.0-C-425-675, 10 m focal length, CVI Laser Optics, UK) was placed in a slider and can be inserted into the emission light path for 3D imaging. Camera and microscope were controlled using the μManager software^42^. Laser intensities for sample excitation ranged between 1 and 5 kW/cm^2^. A preamplifier gain of 3 and an EM-gain of 200 were used to record 10,000–15,000 frames at a frame rate of 33–50 Hz in HILO mode for PAINT/*d*STORM and 10 Hz for PALM imaging. PAINT imaging was performed in PBS or tris/MEA-buffer (100 mM tris pH 8.0, 100 mM MEA, 10 mM NaCl) containing the desired PAINT probes at 100–800 pM concentration. For sequential multi-colour-imaging of the bulk DNA in the same cells (Fig. 1a), the sample was removed from the microscope after imaging and washed at least thrice with PBS before applying the new buffer. Relocation of the sample was performed as described elsewhere^6^. No sample removal was necessary for all other fixed-cell experiments. 150 mM tris pH 8.0 was used for parallel multi-colour experiments including PALM imaging.

### Data analysis

The generated 2D SMLM data was analysed with rapi*d*STORM v3.31^43^ and post-processed with the open-source image analysis platform Fiji^44^. Single-molecule localisations were fitted using free PSF fit parameters and a global threshold of 200 (Hoechst-conjugates), 100 (PALM imaging of RNAP-PAmCherry1) or 50 photons (hydrophobic membrane probes and cytosolic PAmCherry1). Localisations were filtered according to their full width at half maximum (FWHM, <440 nm, 520 nm, 540 nm and 560 nm for membrane probes, Hoechst-JF_503_, Hoechst-JF_549_ and Hoechst-JF_646_, respectively) and PSF symmetry (0.7 < FWHM(x)/FWHM(y) < 1.3). Wavelength dependent chromatic aberrations were corrected using linear correction matrices generated using the “Alignment Fitter” tool implemented in rapidSTORM. For this purpose, calibration measurements were performed, in which sequences of the same surface-immobilised TetraSpeck Microspheres (0.1 µm, Thermo Fisher Scientific) were acquired in each spectral channel (typically 15–30 equally distributed fiducial markers within the imaging ROI). Fiducial marker signals were localised and the resulting localisation files were used to generate the correction matrices.

3D data was analysed using the custom-written software Insight3. Calibration curves were recorded using TetraSpeck Microspheres (z-stack step size 25 nm). Note that robust fitting of orange-emitting molecules is only possible in an axial section of ~600 nm.

Quantitative analyses of photon yields per imaging frame and the experimental localisation precision (NeNA)^45^ were performed without tracking subsequent-frame emissions. In all other cases, a spatiotemporal filter (3σ confidence interval, based on the localisation precision and 2 dark frames) was applied after the initial single-molecule fitting routine. For binding-time analysis, we determined the number of frames a fluorophore emitted light (Fig. S7). 200–400 pM dye either in PBS or tris/MEA buffer were added to fixed and permeabilised *E. coli* cells. Single molecule signals were filtered for PSF width and shape and brightness (>50 photons for Nile Red, >100 photons for all other probes). For each DNA- and membrane-targeted probe, 6 ROIs from 2–3 measurements were analysed and normalized distributions were averaged.

Normalized intensity profiles were generated from super-resolved images that were smoothed with a Gaussian blur of 20 nm using Fiji. An intensity profile was plotted along the longitudinal axis of the bacteria. The line width was set to 200 nm for the membrane images and 800 nm for the PAmCherry1 and DNA images. Resulting intensity profiles were normalised using the software Origin2018 (OriginLab, USA) and the normalised intensities of the respective channels (PAmCherry1, DNA and membrane) were plotted against the distance.

#### Determination of genetic loci positions

z-stacks (100 nm step size) of the GFP-ParB channel were rescaled by a factor of 2 in x/y dimension in order to reduce the pixel size. Background was subtracted using the “Subtract Background” plugin with a rolling ball radius of 20 pixels. Deconvolution was carried out using the Wiener Filter Preconditioned Landweber algorithm with reflexive boundaries, 32-bit float output, and single precision for 20 iterations. For determining the exact position of the genetic loci, the entire deconvolved z-stack was averaged and further processed using rapi*d*STORM v.3.31.

Custom-written Fiji macros were used to reorient, straighten and normalise the bacteria for cell length and width to construct average localisation maps in loci-tagging experiments (Fig. 3).

#### DBSCAN analysis

Noise localisations and low density regions of nascent and bulk DNA were filtered using DBSCAN^35^, implemented in the localisation microscopy analysis software LAMA^46^ For the bulk DNA channel, all localisations with less than 19 neighbours (minpts = 20) within a 50 nm radius were removed from the localisation dataset. For filtering of pulse-labelled DNA, minpts = 7 was applied, a value determined from click-labelled cells lacking EdU (Fig. S13). In order to determine the nucleoid coverage by nascent DNA, a Gaussian filter (σ = 3 pixels) was applied to the DBSCAN filtered image which was subsequently binarised using Otsu’s algorithm. The area of the thresholded regions per cell was finally determined in Fiji. Cluster radii of nascent DNA foci in cells treated with EdU for 2 min were calculated from the area covered by each individual cluster (detected by the DBSCAN algorithm).

#### Counting of single-molecule localisations

The LAMA software package^46^ was used to assign a grayscale value of one per single-molecule localisation to the pixel of its respective position. Cell outlines were generated using the membrane PAINT channel and used to determine the integrated intensity for each single *E. coli* cell, reflecting the number of localisations.

#### Image presentation

A Gaussian blur of 10 nm was applied to all images (except Figs S9 and S10), representing the average localisation precision for SMLM measurements (Table S1). Contrast was adjusted for optimal visualization, unless differences in signal intensities were investigated (Figs S3 and S9).

## Electronic supplementary material

Supplementary information

## Data Availability

The Janelia Fluor and membrane-targeted PAINT dyes are available from Luke Lavis on request. Raw and processed data contributing to this study is available from the corresponding author on request.
