# Supplementary material for: A toolbox for multiplexed super-resolution imaging of the E. coli nucleoid and membrane using novel PAINT labels
Source: Sci Rep. 2018 Oct 3;8:14768. doi: 10.1038/s41598-018-33052-3 (PMC6170473; doi:10.1038/s41598-018-33052-3)
Supplement: Supplementary file 1 — Supplementary information [file 41598_2018_33052_MOESM1_ESM.pdf]

A toolbox for multiplexed super-resolution imaging of the *E. coli* nucleoid and membrane using novel PAINT labels

Christoph K. Spahn[1,*], Mathilda Glaesmann[1,*], Jonathan B. Grimm[2], Anthony X. Ayala[2], Luke D. Lavis[2]†, Mike Heilemann[1]†

[1]Institute of Physical and Theoretical Chemistry, Goethe-University Frankfurt, Max-von-Laue-Str. 7, 60438 Frankfurt, Germany

[2]Janelia Research Campus, Howard Hughes Medical Institute, 19700 Helix Drive, Ashburn, Virginia 20147, USA.

[*] These authors contributed equally to this work.

† Corresponding authors

Supplementary Material

General Experimental Information for Synthesis of PAINT Labels

Commercial reagents were obtained from reputable suppliers and used as received. All solvents were purchased in septum-sealed bottles stored under an inert atmosphere. All reactions were sealed with septa through which a nitrogen atmosphere was introduced unless otherwise noted. Reactions were conducted in round-bottomed flasks or septum-capped crimp-top vials containing Teflon-coated magnetic stir bars. Heating of reactions was accomplished with a silicon oil bath or an aluminium reaction block on top of a stirring hotplate equipped with an electronic contact thermometer to maintain the indicated temperatures.

Reactions were monitored by thin layer chromatography (TLC) on precoated TLC glass plates (silica gel 60 F_{254}, 250 µm thickness) or by LC/MS (Phenomenex Kinetex 2.1 mm × 30 mm 2.6 µm C18 column; 5 µL injection; 5–98% MeCN/H_2O, linear gradient, with constant 0.1% v/v HCO_2H additive; 6 min run; 0.5 mL/min flow; ESI; positive ion mode). TLC chromatograms were visualised by UV illumination or developed with *p*-anisaldehyde, ceric ammonium molybdate, or $KMnO_4$ stain. Reaction products were purified by flash chromatography on an automated purification system using pre-packed silica gel columns or by preparative HPLC (Phenomenex Gemini–NX 30 × 150 mm 5 µm C18 column). Analytical HPLC analysis was performed with an Agilent Eclipse XDB 4.6 × 150 mm 5 µm C18 column

under the indicated conditions. High-resolution mass spectrometry was performed by the High Resolution Mass Spectrometry Facility at the University of Iowa.

NMR spectra were recorded on a 400 MHz spectrometer. ^{1}H and ^{13}C chemical shifts were referenced to TMS or residual solvent peaks. Data for ^{1}H NMR spectra are reported as follows: chemical shift (δ ppm), multiplicity (s = singlet, d = doublet, t = triplet, q = quartet, dd = doublet of doublets, m = multiplet), coupling constant (Hz), integration. Data for ^{13}C NMR spectra are reported by chemical shift (δ ppm) with hydrogen multiplicity (C, CH, CH_2, CH_3) information obtained from DEPT spectra.

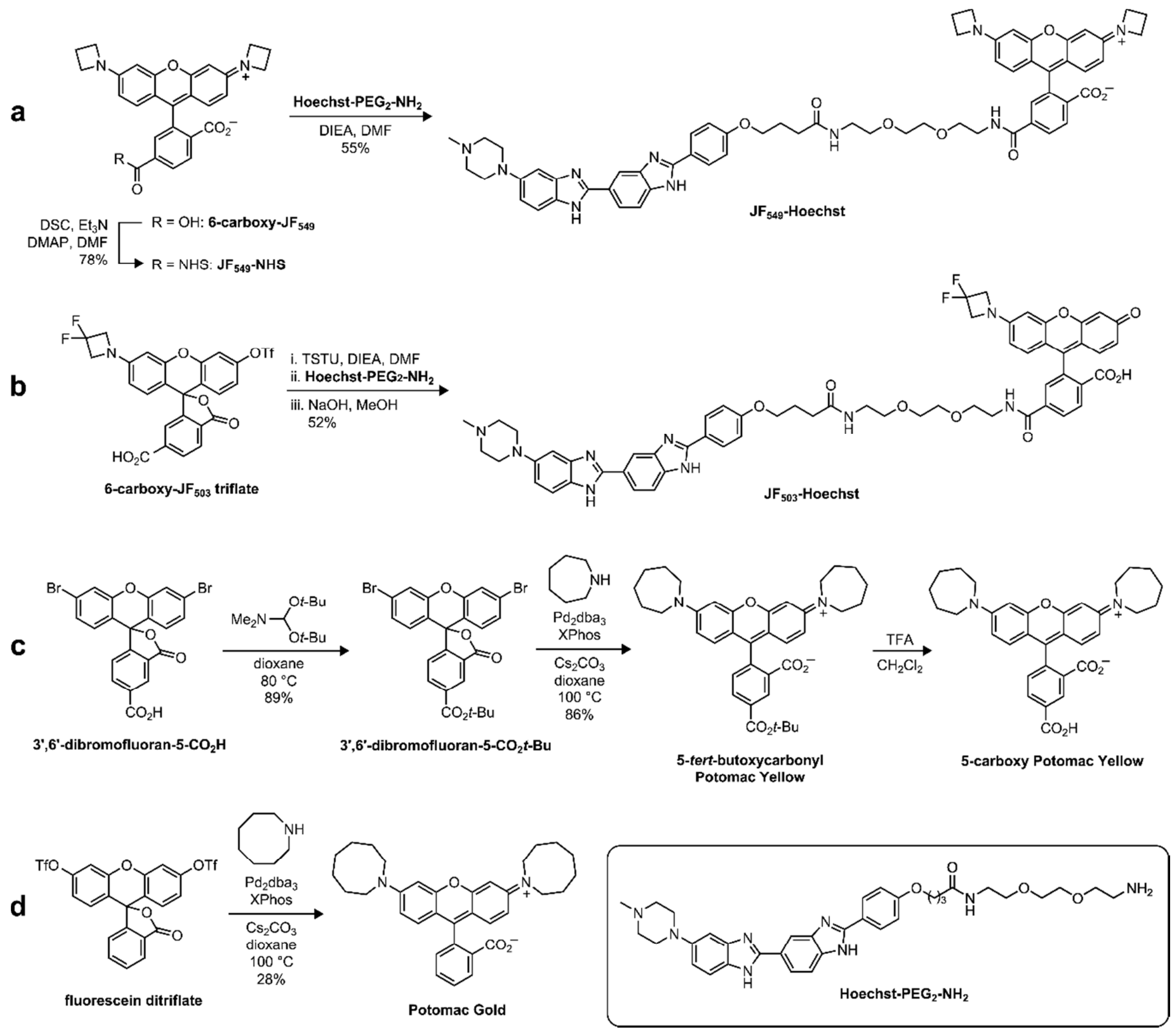

Figure S1. Synthetic routes to (a) JF_{549}-Hoechst, (b) JF_{503}-Hoechst, (c) 5-carboxy Potomac Yellow, and (d) Potomac Gold.

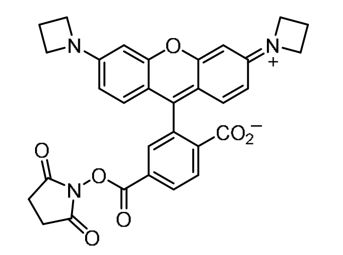

JF_{549}-NHS: 6-Carboxy-JF_{549}[1] (20 mg, 35.2 µmol) was combined with DSC (19.8 mg, 77.4 µmol, 2.2 eq) in DMF (1.5 mL). After adding Et_3N (14.7 µL, 106 µmol, 3 eq) and DMAP (0.4 mg, 3.52 µmol, 0.1 eq), the reaction was stirred at room temperature for 2 h. Purification of the crude reaction mixture by reverse phase HPLC (10–95% MeCN/H_2O, linear gradient, with constant 0.1% v/v TFA additive) afforded 18.3 mg (78%, TFA salt) of the title compound as a dark purple solid. ^1H NMR (DMSO-d_6, 400 MHz) δ 8.47 (dd, J = 8.2, 1.8 Hz, 1H), 8.42 (d, J = 8.3 Hz, 1H), 8.12 (d, J = 1.7 Hz, 1H), 7.06 (d, J = 9.2 Hz, 2H), 6.61 (dd, J = 9.1, 2.2 Hz, 2H), 6.56 (d, J = 2.2 Hz, 2H), 4.26 (t, J = 7.7 Hz, 8H), 2.90 (s, 4H), 2.45 (p, J = 7.6 Hz, 4H); Analytical HPLC: t_R =11.7 min, 97.4% purity (5 µL injection; 10–95% MeCN/H_2O, linear gradient, with constant 0.1% v/v TFA additive; 20 min run; 1 mL/min flow; ESI; positive ion mode; detection at 550 nm); MS (ESI) calcd for $C_{31}H_{26}N_3O_7$ $[M+H]^+$ 552.2, found 552.0.

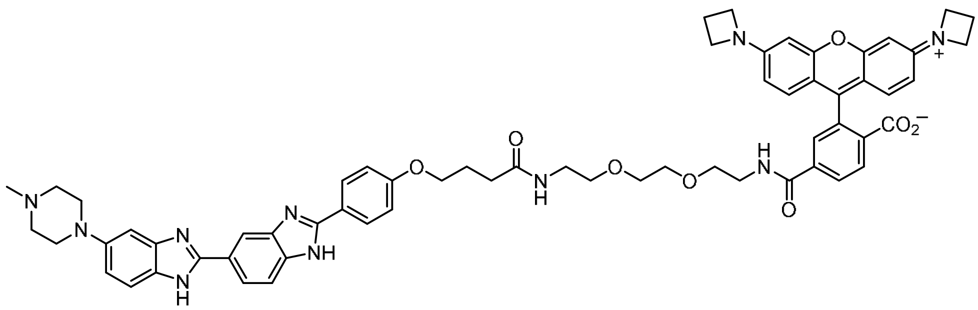

JF_{549}–Hoechst: JF_{549}-NHS (10 mg, 15.0 µmol) and Hoechst-PEG$_2$-NH$_2$[2] (4·TFA salt; 19.8 mg, 18.0 µmol, 1.2 eq) were combined in DMF (1 mL), and DIEA (26.2 µL, 150 µmol, 10 eq) was added. After stirring the reaction at room temperature for 1 h, the crude reaction mixture was directly purified by reverse phase HPLC (10–50% MeCN/H_2O, linear gradient, with constant 0.1% v/v TFA additive) to afford 12.7 mg (55%, 4·TFA salt) of the title compound as a purple solid. ^1H NMR (CD_3OD, 400 MHz) δ 8.37 (d, J = 8.3 Hz, 1H), 8.34 – 8.30 (m, 1H), 8.20 (dd, J = 8.2, 1.8 Hz, 1H), 8.05 – 8.00 (m, 2H), 8.01 (dd, J = 8.5, 1.8 Hz, 1H), 7.88 (d, J = 1.7 Hz, 1H), 7.86 – 7.82 (m, 1H), 7.72 (d, J = 9.1 Hz, 1H), 7.39 (dd, J = 9.0, 2.3 Hz, 1H), 7.32 (d, J = 2.1 Hz, 1H), 7.11 – 7.05 (m, 2H), 6.98 (d, J = 9.2 Hz, 2H), 6.46 (dd, J = 9.2, 2.2 Hz, 2H), 6.36 (d, J = 2.2 Hz, 2H), 4.18 (t, J = 7.7 Hz, 8H), 4.04 (t, J = 6.3 Hz, 2H), 4.00 – 3.34 (m, 20H), 3.02 (s, 3H), 2.47 (p, J = 7.6 Hz, 4H), 2.38 (t, J = 7.2 Hz, 2H), 2.07 (p, J = 6.5 Hz, 2H); Analytical HPLC: t_R = 12.5 min, >99% purity (5 µL injection; 10–50% MeCN/H_2O, linear gradient, with constant 0.1% v/v TFA additive; 20 min run; 1 mL/min flow; ESI; positive ion

mode; detection at 550 nm); HRMS (ESI) calcd for $C_{62}H_{65}N_{10}O_8$ $[M+H]^+$ 1077.4981, found 1077.4988.

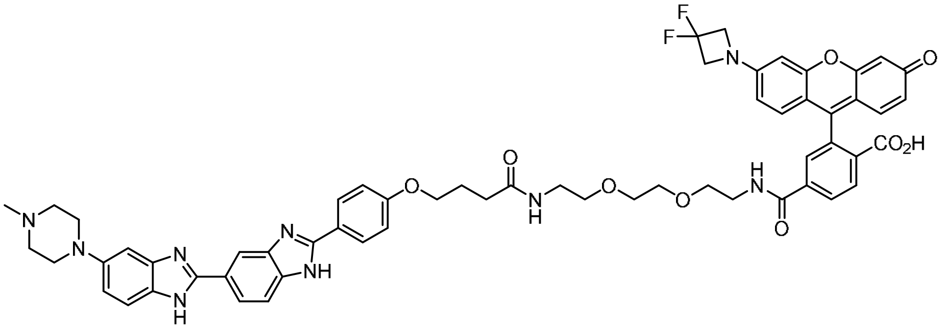

JF$_{503}$–Hoechst: 6-Carboxy-JF$_{503}$ triflate[3] (13.6 mg, 19.4 μmol) was combined with TSTU (12.9 mg, 4.29 μmol, 2.2 eq) in DMF (2.0 mL). After adding DIEA (34.0 μL, 195 μmol, 10 eq), the reaction was stirred at room temperature for 1 h. Hoechst-PEG$_2$-NH$_2$ (4·TFA salt; 51.3 mg, 46.8 μmol, 2.4 eq) was then added, and the reaction was stirred for 72 h at room temperature. After adding MeOH (1.0 mL) and 1 N NaOH (300 μL) and stirring an additional 24 h at room temperature, the reaction was acidified with 1 N HCl (350 μL) and concentrated to dryness. The crude material was purified by reverse phase HPLC (10–50% MeCN/H_2O, linear gradient, with constant 0.1% v/v TFA additive) to provide 15.5 mg (52%, 4·TFA salt) of the title compound as an orange solid. ^{1}H NMR (CD_3OD, 400 MHz) δ 8.37 (d, *J* = 1.7 Hz, 1H), 8.25 (d, *J* = 8.1 Hz, 1H), 8.19 (dd, *J* = 8.2, 1.6 Hz, 1H), 8.09 – 8.04 (m, 2H), 8.03 (dd, *J* = 8.5, 1.7 Hz, 1H), 7.88 (d, *J* = 8.6 Hz, 1H), 7.80 – 7.77 (m, 1H), 7.72 (d, *J* = 9.0 Hz, 1H), 7.40 (dd, *J* = 9.1, 2.1 Hz, 1H), 7.31 (d, *J* = 2.1 Hz, 1H), 7.15 – 7.08 (m, 2H), 6.98 (d, *J* = 8.9 Hz, 1H), 6.95 (d, *J* = 8.9 Hz, 1H), 6.91 (d, *J* = 2.3 Hz, 1H), 6.78 (dd, *J* = 8.9, 2.3 Hz, 1H), 6.63 (d, *J* = 2.2 Hz, 1H), 6.58 (dd, *J* = 9.0, 2.2 Hz, 1H), 4.51 (t, *J* = 11.7 Hz, 4H), 4.08 (t, *J* = 6.2 Hz, 2H), 4.04 – 3.85 (m, 2H), 3.73 – 3.51 (m, 10H), 3.48 (t, *J* = 5.4 Hz, 2H), 3.43 – 3.16 (m, 6H), 3.02 (s, 3H), 2.39 (t, *J* = 7.3 Hz, 2H), 2.08 (p, *J* = 6.6 Hz, 2H); Analytical HPLC: t_R = 8.2 min, >99% purity (10–95% MeCN/H_2O, linear gradient, with constant 0.1% v/v TFA additive; 20 min run; 1 mL/min flow; ESI; positive ion mode; detection at 500 nm); MS (ESI) calcd for $C_{59}H_{58}F_2N_9O_9$ $[M+H]^+$ 1074.4, found 1073.9.

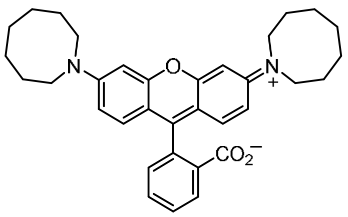

Potomac Gold: A vial was charged with fluorescein ditriflate[4] (275 mg, 0.461 mmol), Pd_2dba_3 (42 mg, 46.1 μmol, 0.1 eq), XPhos (66 mg, 0.138 mmol, 0.3 eq), and Cs_2CO_3 (421 mg, 1.29 mmol, 2.8 eq). The vial was sealed and evacuated/backfilled with nitrogen (3×).

Dioxane (3 mL) was added, and the reaction was flushed again with nitrogen (3×). Following the addition of heptamethyleneimine (140 μL, 1.11 mmol, 2.4 eq), the reaction was stirred at 100 °C for 4 h. It was then cooled to room temperature, diluted with MeOH, deposited onto Celite, and concentrated to dryness. Purification by silica gel chromatography (0–10% MeOH (2 M NH_3)/CH_2Cl_2, linear gradient; dry load on Celite) afforded the title compound (68 mg, 28%) as a purple-pink solid. ^1H NMR ($CDCl_3$, 400 MHz) δ 8.00 (dt, *J* = 7.4, 1.0 Hz, 1H), 7.63 (td, *J* = 7.4, 1.3 Hz, 1H), 7.57 (td, *J* = 7.4, 1.1 Hz, 1H), 7.22 (dt, *J* = 7.6, 1.0 Hz, 1H), 6.57 (d, *J* = 8.9 Hz, 2H), 6.46 (d, *J* = 2.6 Hz, 2H), 6.35 (dd, *J* = 8.9, 2.6 Hz, 2H), 3.51 – 3.38 (m, 8H), 1.80 – 1.69 (m, 8H), 1.64 – 1.48 (m, 12H); ^{13}C NMR ($CDCl_3$, 101 MHz) δ 170.0 (C), 153.5 (C), 152.8 (C), 150.2 (C), 134.4 (CH), 129.3 (CH), 129.0 (CH), 128.3 (C), 125.0 (CH), 124.4 (CH), 108.1 (CH), 106.2 (C), 97.7 (CH), 51.1 (CH_2), 27.2 (CH_2), 27.1 (CH_2), 26.8 (CH_2); Analytical HPLC: t_R = 10.8 min, >99% purity (5 μL injection; 10–95% MeCN/H_2O, linear gradient, with constant 0.1% v/v TFA additive; 20 min run; 1 mL/min flow; ESI; positive ion mode; detection at 550 nm); MS (ESI) calcd for $C_{34}H_{39}N_2O_3$ $[M+H]^+$ 523.3, found 523.3.

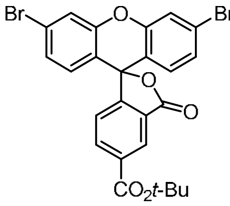

3′,6′-Dibromofluoran-5-CO₂*t*-Bu: A suspension of 3′,6′-dibromofluoran-5-CO_2H[5] (1.50 g, 2.99 mmol) in dioxane (10 mL) was heated to 80 °C, and *N*,*N*-dimethylformamide di-*tert*-butyl acetal (2.87 mL, 11.95 mmol, 4 eq) was added dropwise over 5 min. The reaction was stirred at 80 °C for 30 min. Additional *N*,*N*-dimethylformamide di-*tert*-butyl acetal (2.87 mL, 11.95 mmol, 4 eq) was then added in the same manner as before. After stirring for another 15 min at 80 °C, the reaction was cooled to room temperature, diluted with saturated $NaHCO_3$, and extracted with EtOAc (2×). The combined organic extracts were dried over anhydrous $MgSO_4$, filtered, and concentrated *in vacuo*. Flash chromatography on silica gel (0–10% EtOAc/toluene, linear gradient) provided the title compound as a white solid (1.49 g, 89%). ^1H NMR ($CDCl_3$, 400 MHz) δ 8.62 (dd, *J* = 1.5, 0.8 Hz, 1H), 8.30 (dd, *J* = 8.0, 1.5 Hz, 1H), 7.51 (d, *J* = 1.9 Hz, 2H), 7.19 (dd, *J* = 8.5, 1.9 Hz, 2H), 7.17 (dd, *J* = 8.0, 0.7 Hz, 1H), 6.68 (d, *J* = 8.5 Hz, 2H), 1.63 (s, 9H); ^{13}C NMR ($CDCl_3$, 101 MHz) δ 168.3 (C), 163.9 (C), 156.1 (C), 151.2 (C), 136.6 (CH), 134.8 (C), 129.2 (CH), 127.8 (CH), 127.0 (CH), 126.2 (C), 124.6 (C), 123.9 (CH), 120.7 (CH), 117.5 (C), 82.8 (C), 81.4 (C), 28.3 (CH_3); MS (ESI) calcd for $C_{25}H_{19}Br_2O_5$ $[M+H]^+$ 557.0/559.0/561.0, found 557.0/559.0/561.0.

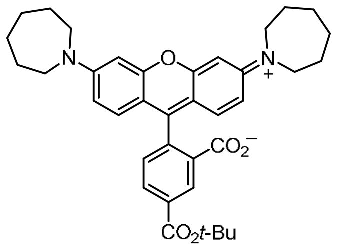

5-*tert*-Butoxycarbonyl Potomac Yellow: A vial was charged with 3′,6′-dibromofluoran-5-CO_2*t*-Bu (500 mg, 0.896 mmol), Pd_2dba_3 (82 mg, 89.6 μmol, 0.1 eq), XPhos (128 mg, 0.269 mmol, 0.3 eq), and Cs_2CO_3 (817 mg, 2.51 mmol, 2.8 eq). The vial was sealed and evacuated/backfilled with nitrogen (3×). Dioxane (6 mL) was added, and the reaction was flushed again with nitrogen (3×). Following the addition of hexamethyleneimine (242 μL, 2.15 mmol, 2.4 eq), the reaction was stirred at 100 °C for 4 h. It was then cooled to room temperature, diluted with MeOH, deposited onto Celite, and concentrated to dryness. Purification by silica gel chromatography (0–10% MeOH (2 M NH_3)/CH_2Cl_2, linear gradient; dry load on Celite) afforded 457 mg (86%) of the title compound as a purple solid. ^{1}H NMR ($CDCl_3$, 400 MHz) δ 8.59 (dd, *J* = 1.6, 0.7 Hz, 1H), 8.26 (dd, *J* = 8.0, 1.5 Hz, 1H), 7.25 (dd, *J* = 8.0, 0.6 Hz, 1H), 6.53 (d, *J* = 8.9 Hz, 2H), 6.46 (d, *J* = 2.5 Hz, 2H), 6.35 (dd, *J* = 8.9, 2.6 Hz, 2H), 3.49 – 3.41 (m, 8H), 1.82 – 1.73 (m, 8H), 1.64 (s, 9H), 1.58 – 1.50 (m, 8H); ^{13}C NMR ($CDCl_3$, 101 MHz) δ 169.2 (C), 164.6 (C), 156.4 (C), 153.5 (C), 151.0 (C), 135.4 (CH), 133.7 (C), 129.1 (CH), 128.5 (C), 126.5 (CH), 124.5 (CH), 108.1 (CH), 105.6 (C), 97.6 (CH), 82.2 (C), 49.5 (CH_2), 28.4 (CH_3), 27.6 (CH_2), 27.1 (CH_2); Analytical HPLC: t_R = 11.8 min, 97.8% purity (5 μL injection; 10–95% MeCN/H_2O, linear gradient, with constant 0.1% v/v TFA additive; 20 min run; 1 mL/min flow; ESI; positive ion mode; detection at 550 nm); MS (ESI) calcd for $C_{37}H_{43}N_2O_5$ $[M+H]^+$ 595.3, found 595.3.

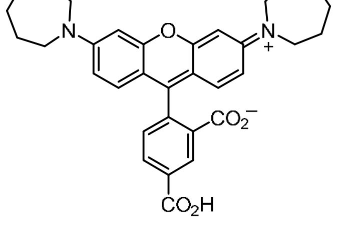

5-Carboxy Potomac Yellow: 5-*tert*-Butoxycarbonyl Potomac Yellow (296 mg, 0.498 mmol) was taken up in CH_2Cl_2 (5 mL), and trifluoroacetic acid (1 mL) was added. The reaction was stirred at room temperature for 6 h. Toluene (5 mL) was added; the reaction mixture was concentrated to dryness and then azeotroped with MeOH three times to provide the title compound as a dark pink solid (TFA salt, 321 mg, 99%). Analytical HPLC and NMR indicated that the material was >95% pure. An analytically pure (>99%) sample was obtained through further purification by reverse phase HPLC (10–95% MeCN/H_2O, linear gradient, with constant 0.1% v/v TFA additive); the HPLC-purified batch was used for all characterization

and imaging purposes. ^{1}H NMR (CD_3OD, 400 MHz) δ 8.92 (d, *J* = 1.7 Hz, 1H), 8.43 (dd, *J* = 7.9, 1.7 Hz, 1H), 7.54 (d, *J* = 7.9 Hz, 1H), 7.12 (d, *J* = 9.5 Hz, 2H), 7.07 (dd, *J* = 9.5, 2.3 Hz, 2H), 7.00 (d, *J* = 2.3 Hz, 2H), 3.79 (t, *J* = 6.1 Hz, 8H), 1.98 – 1.78 (m, 8H), 1.68 – 1.54 (m, 8H); ^{13}C NMR (CD_3OD, 101 MHz) δ 167.9 (C), 167.3 (C), 159.8 (C), 159.3 (C), 158.1 (C), 139.5 (C), 134.5 (CH), 134.2 (C), 133.5 (CH), 132.8 (C), 132.2 (CH), 132.1 (CH), 115.5 (CH), 114.7 (C), 97.3 (CH), 52.0 (CH_2), 27.9 (CH_2), 27.4 (CH_2); Analytical HPLC: t_R = 10.6 min, 97.0% purity (5 µL injection; 10–95% MeCN/H_2O, linear gradient, with constant 0.1% v/v TFA additive; 20 min run; 1 mL/min flow; ESI; positive ion mode; detection at 550 nm); MS (ESI) calcd for $C_{33}H_{35}N_2O_5$ $[M+H]^+$ 539.3, found 539.2.

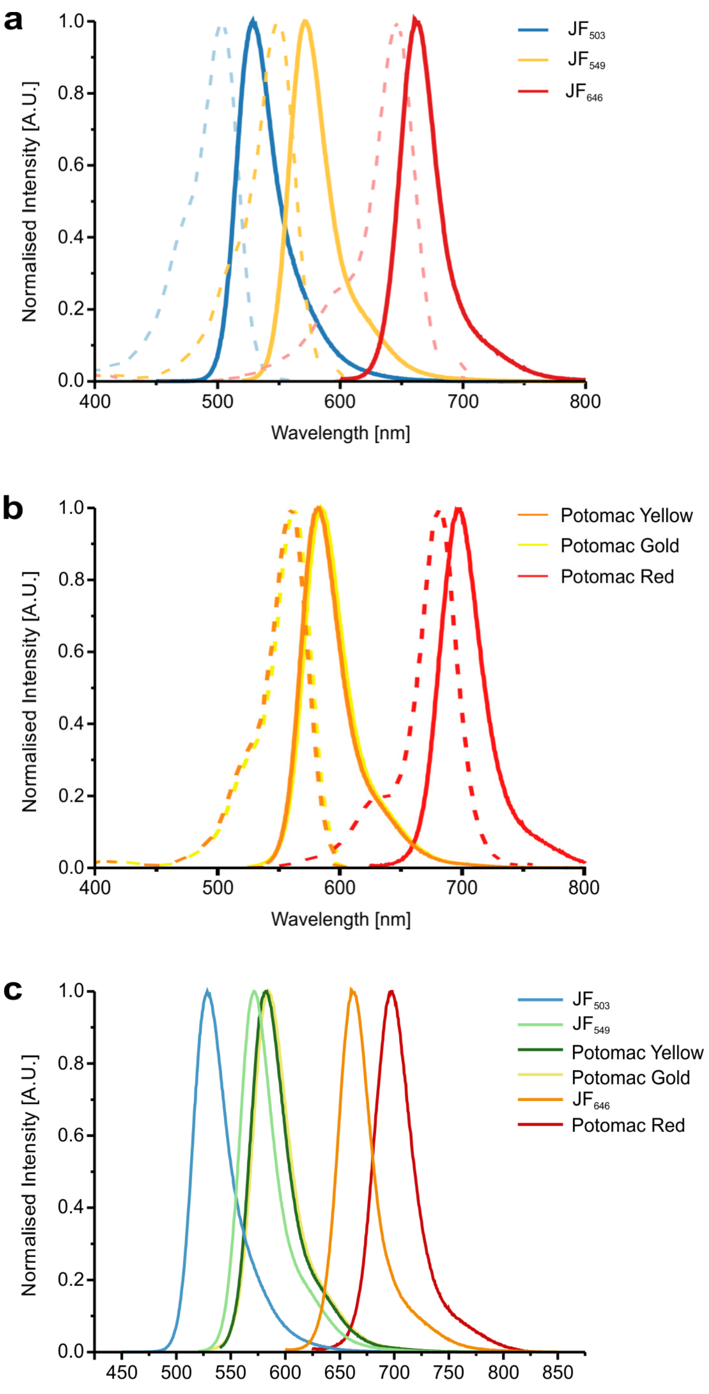

Figure S2: Fluorescence spectra of JF and Potomac dyes. **a**, Fluorescence excitation (dashed line) and emission (solid line) spectra of parent JF_{503}, JF_{549}, and JF_{646} dyes that were used in the Hoechst conjugates. **b**, Fluorescence spectra of Potomac Yellow, Potomac Gold and Potomac Red dyes, the excitation is shown in dashed lines and the emission is solid lines. **c**, Fluorescence emission spectra of JF_{503}, JF_{549}, JF_{646}, Potomac Yellow, Potomac Gold and Potomac Red**.**

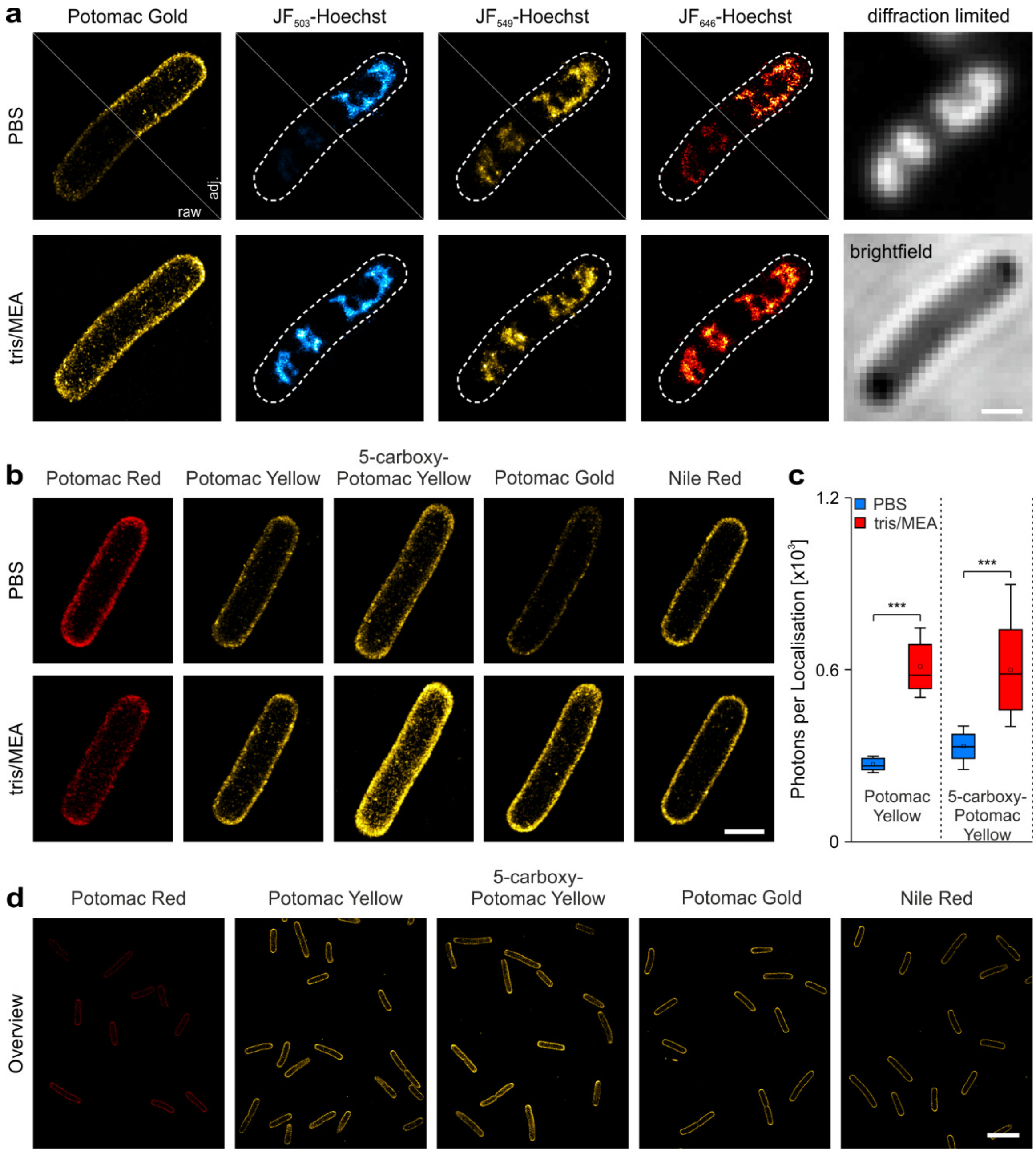

Figure S3: Super-resolution imaging of the bacterial nucleoid and membrane using PAINT dyes in different imaging buffers. **a,** PAINT images of the nucleoid of a fast-grown *E. coli* cell labelled with different Hoechst-conjugated rhodamine dyes and imaged in PBS (top panel) or tris/MEA (bottom panel). For comparison, the membrane was imaged using the hydrophobic PAINT probe Potomac Gold (left column), cell outlines are indicated as dashed lines. For cells imaged in PBS, brightness and contrast were kept identical in the lower left image in order to compare their performance to tris/MEA (bottom panel). Diffraction limited fluorescence (right panel, top) and brightfield (right panel, bottom) images are shown for comparison (scale bar 1 µm). **b,** PAINT images of the membrane of fast-grown *E. coli* cells, visualised using hydrophobic PAINT probes in PBS (top panel) and tris/MEA (bottom panel). For comparison, brightness and contrast were kept identical in each image. Potomac Yellow and 5-carboxy-Potomac Yellow are also well suited to resolve the bacterial membrane, yet were excluded from Fig. 1 due to fluorescence emission in the same spectral channel as Potomac Gold (scale bar 1 µm). **c,** Brightness of Potomac Yellow and 5-carboxy-Potomac Yellow in PBS (blue

boxes) and tris/MEA buffer (red boxes) determined from single-molecule localisations. Statistical significance was assessed using two tailed, non–parametric Mann–Whitney test: n.s. $P>0.05$; $^{**}P<0.01$;*** $P<0.001$. Rectangles represent mean values and boxes the respective standard deviations. The median is indicated by horizontal lines, while whiskers indicate the minimal and maximal values. **d**, Large regions of interest showing the homogeneous labeling provided by the applied membrane dyes (scale bar: 5 µm).

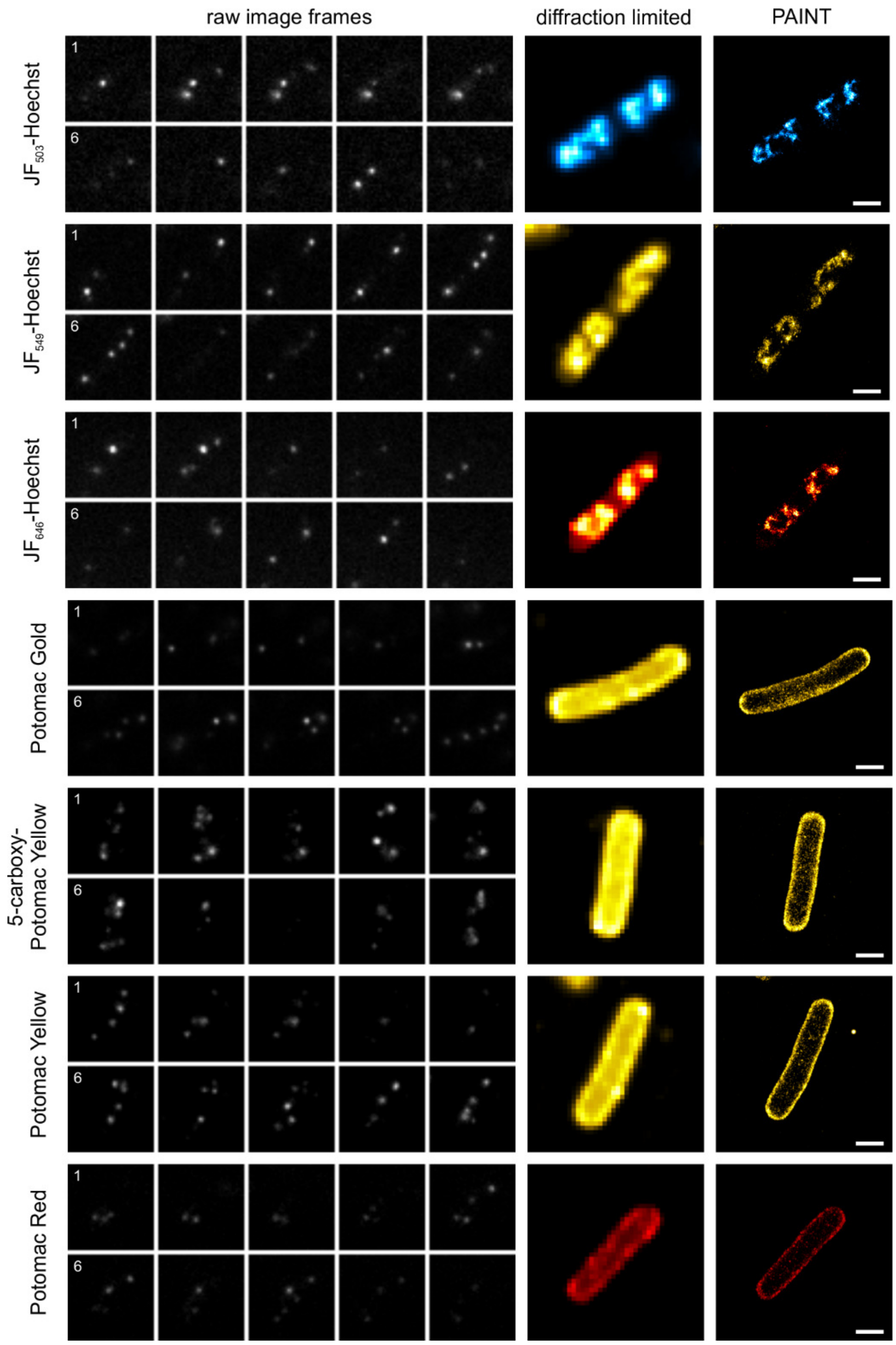

Figure S4: Raw blinking data of DNA- and membrane-targeted PAINT probes. The left column shows ten consecutive frames for each PAINT dye. The respective diffraction limited (standard deviation image of 5,000 imaging frames) and reconstructed PAINT images are shown on the right (scale bar: 1 µm).

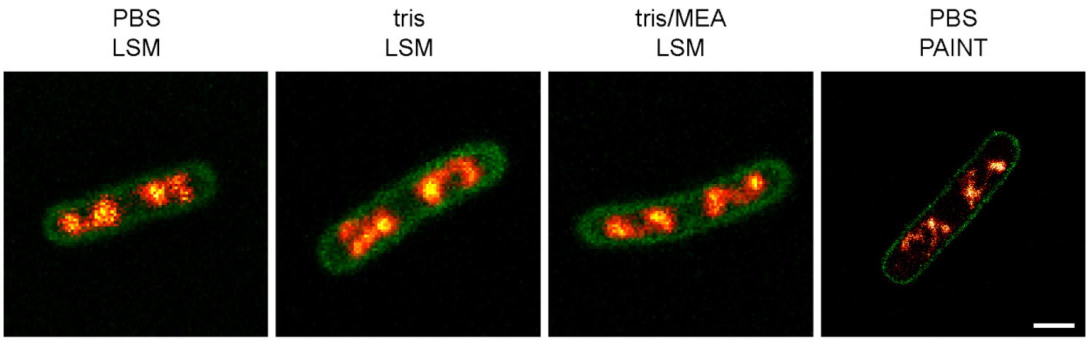

Figure S5: Comparison of dual-colour PAINT imaging with confocal laser scanning microscopy (CLSM). CLSM images of the membrane and nucleoid in fast-grown *E. coli* cells (LB medium, 32°C) labelled with Nile Red and JF646-Hoechst and imaged in either PBS, tris buffer or tris/MEA buffer. To emphasise the resolution improvement provided by PAINT, a super-resolution image of the DNA and membrane acquired in PBS is shown on the right (scale bar: 1 µm).

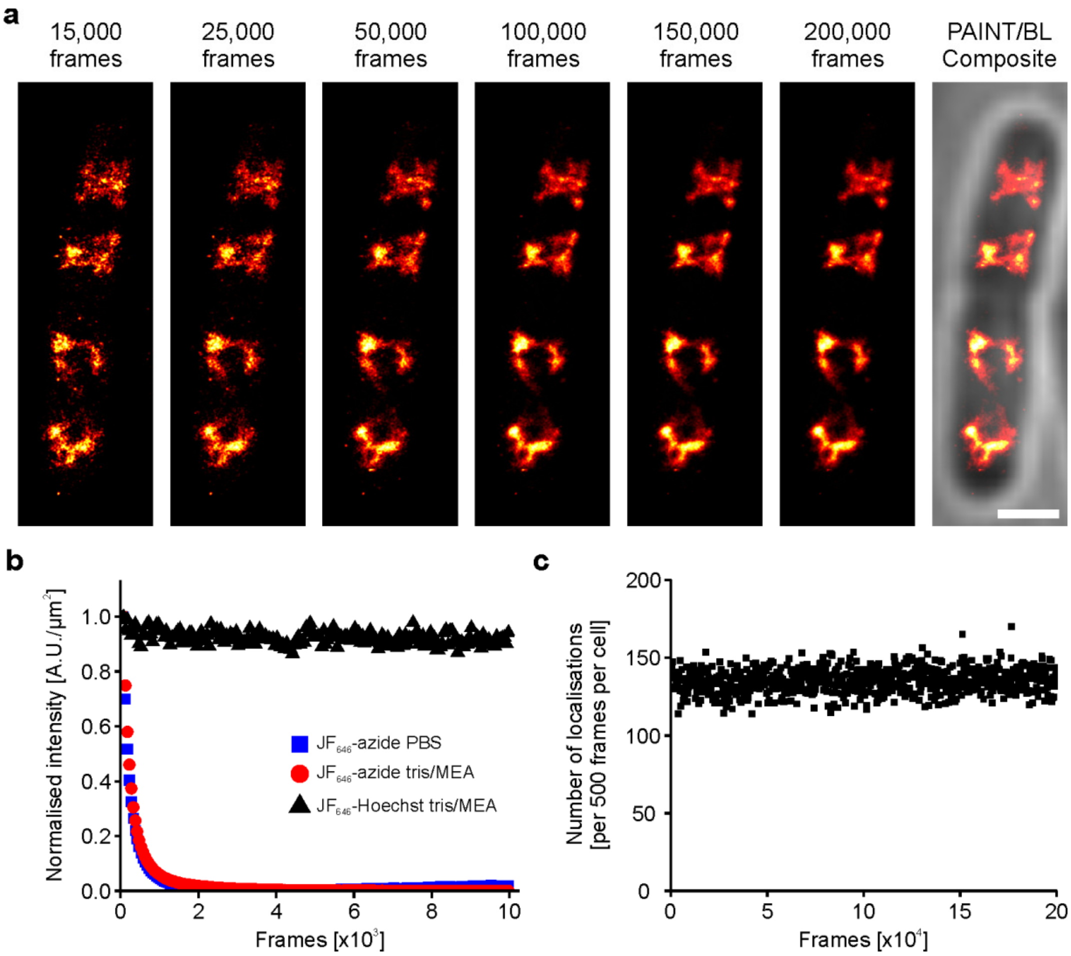

Figure S6: Long time PAINT measurement of the *E. coli* nucleoid using 400 pM JF_{646}-Hoechst. **a**, 200,000 imaging frames were recorded at 50 Hz framerate. Although label density increases with the number of imaging frames acquired, the nucleoid structure does not change substantially and can already be deduced after 15,000 - 25,000 imaging frames (scale bar: 1 µm). **b**, Comparison of the fluorescence signal over time provided by covalently bound JF_{646} (click approach) and the PAINT probe JF_{646}-Hoechst. A decrease in intensity independent from the imaging buffer could be clearly observed for click-labelled JF_{646}. In contrast, the intensity of the JF_{646}-Hoechst PAINT signal remained constant over time.

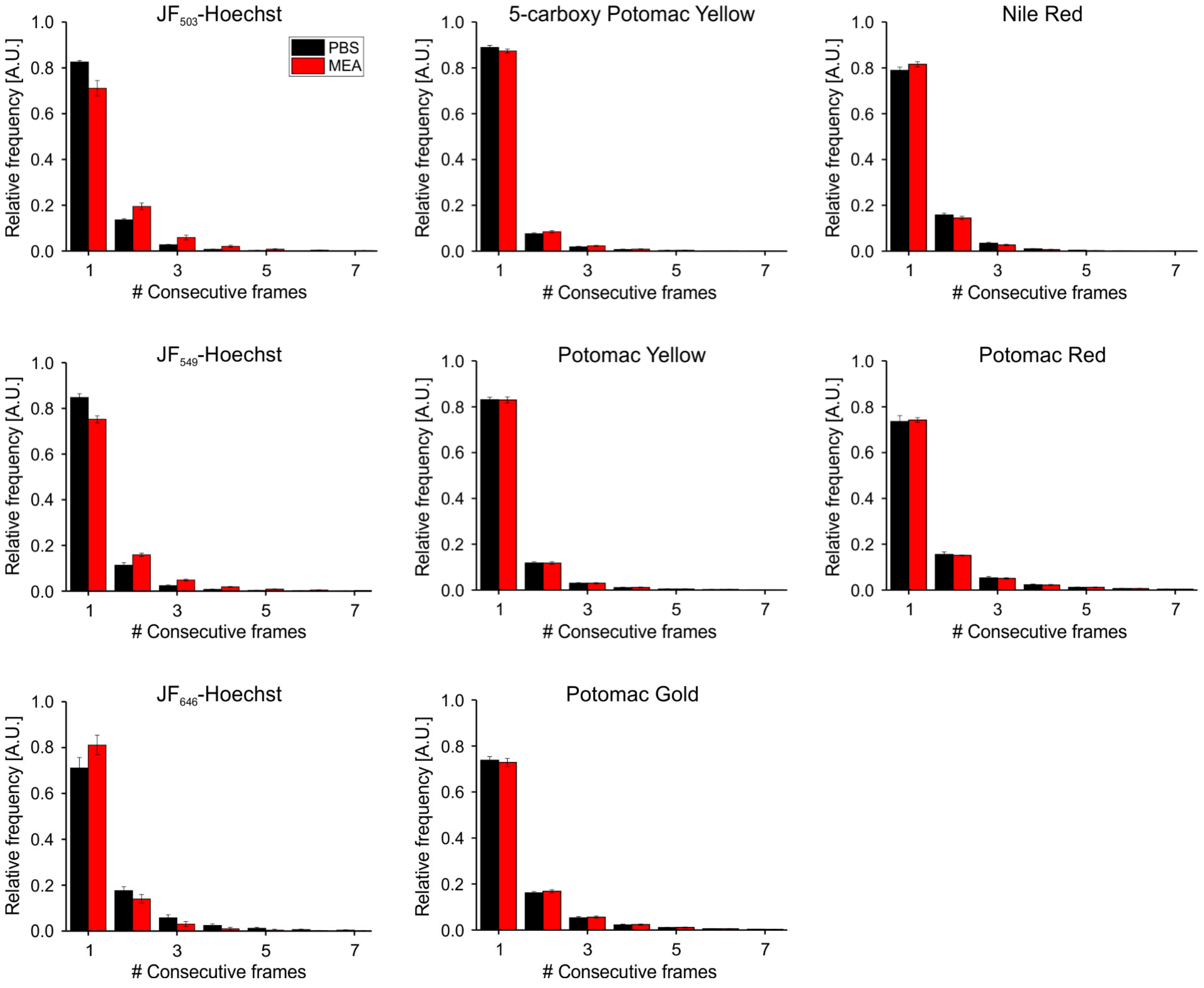

Figure S7: Fluorescence ON times of PAINT fluorophores. The number of (consecutive) frames (20 ms) in which a single fluorophores was detected is histogrammed, and reports on the ON time. In total, 6 regions of interest that were selected in 2-3 independent measurements were analysed. All probes show similar distributions for both applied buffers, indicating that there is likely no additional photoswitching at this time scale. Note that the actual ON times for the PAINT probes are underestimated, since the emission spots of single fluorophores may overlap and since the membrane probes diffuse in the membrane. Bars represent mean values and error bars the respective standard deviation.

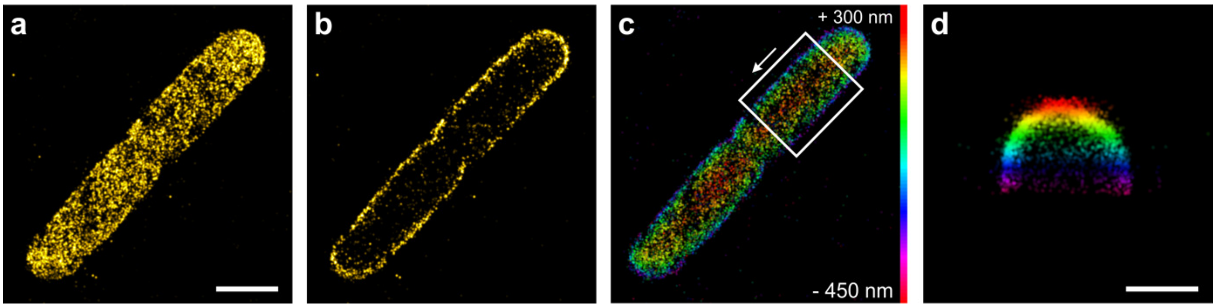

Figure S8: 3D PAINT imaging using the membrane stain Potomac Gold. **a,** 2D projection of astigmatic 3D-SMLM measurement (scale bar: 1 µm). **b,** Bacterial cross-section, generated by filtering the localisations according to their relative axial position. **c,** 3D-rendered image of **a**. The colour indicates the axial position of each single-molecule localisation. **d,** Cross-sectional view of the region of interest shown in c (white rectangle) (scale bar: 500 nm).

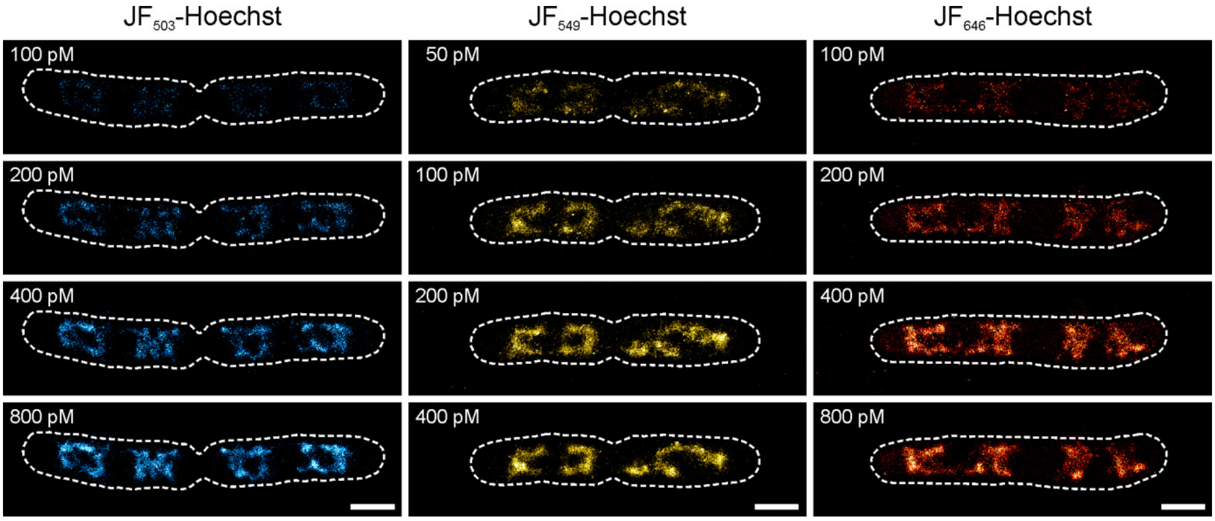

Figure S9: Titration of DNA binding PAINT probes for optimal resolution in SMLM experiments. To avoid sparse labelling or imaging artefacts, nucleoids of fixed MG1655 WT cells (grown in LB medium, 32°C) were resolved using increasing concentrations of the PAINT probes (tris/MEA buffer). The membrane was visualised with Potomac Gold (green) and used to outline cell boundaries in DNA PAINT images (dashed white lines) (scale bars: 1 µm).

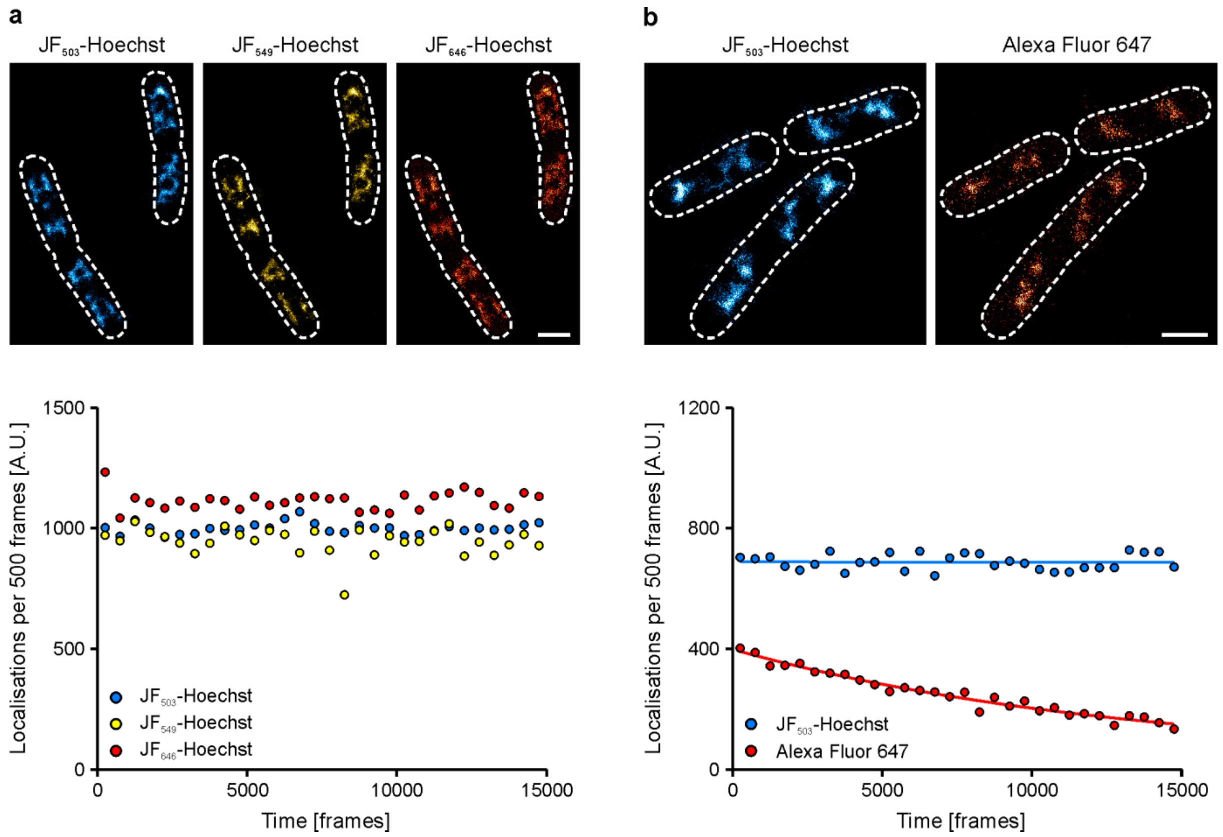

Figure S10: DNA binding probes show reversible binding and constant exchange. **a,** The number of localisations per cell (integrated over 500 frames) remains constant for the imaging period for all PAINT DNA dyes. **b,** In contrast to the PAINT probes (here: JF_{503}-Hoechst), *d*STORM imaging of click-labelled DNA (here: Alexa Fluor 647) shows a decrease in localisations over time due to photobleaching. The bleaching curve can be fitted with a mono-exponential decay function, whereas for JF_{503}-Hoechst, the curve is approximated with a linear function (scale bars: 1 µm).

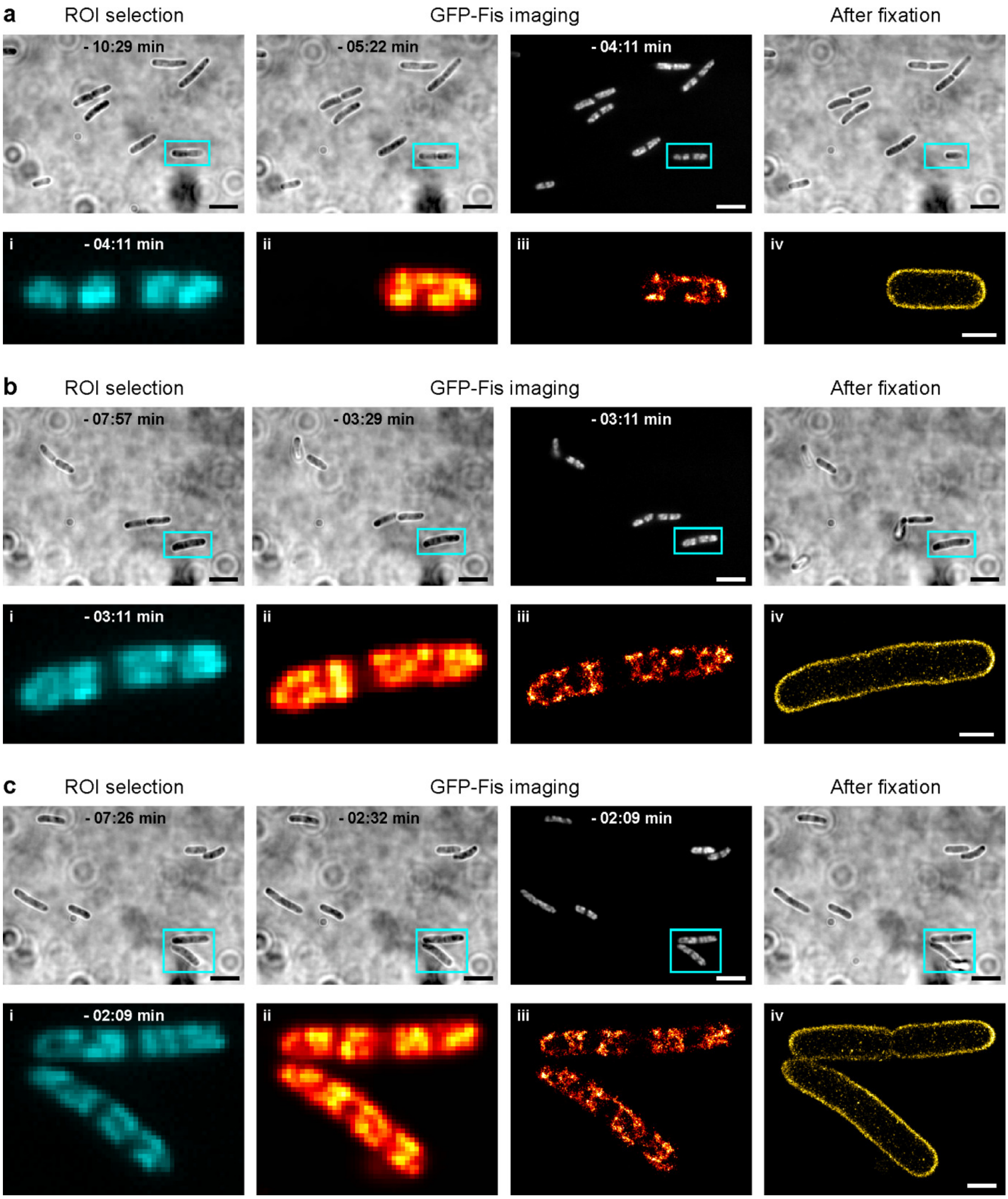

Figure S11: Influence of formaldehyde fixation on nucleoid structure. Living MG1655 wildtype cells grown in LB medium at 32°C and expressing GFP-Fis from an inducible plasmid were immobilised on chitosan-treated surfaces. Nucleoid structures in living cells were recorded **a,** ~ 4 min, **b**, ~3 min and **c**, ~ 2 min prior to formaldehyde fixation. Additionally, brightfield images were taken at different time points before and after fixation (upper panel), demonstrating that immobilised bacteria maintained cell growth and division. The cell marked by the cyan rectangle is used for the comparison of the live-cell GFP-Fis signal with the signal provided by PAINT imaging using JF_{646}-Hoechst. Shown are (i) the diffraction limited image of GFP-Fis prior to fixation, (ii) a diffraction limited image of the PAINT

measurement, reconstructed by calculating the standard deviation image from 5,000 imaging frames, (iii) the respective super-resolved PAINT image acquired with JF_{646}-Hoechst after 30 min FA fixation and (iv) the membrane visualised using Nile Red. Global positioning of the nucleoid is stable over several minutes and nucleoid structure changes only slightly during the 2 - 4 min time difference between GFP-Fis acquisition and chemical fixation. Note that the cell shown in **a** divided within the 4 min time interval (scale bars: 3 µm in the upper panels and 1 µm in the lower panels).

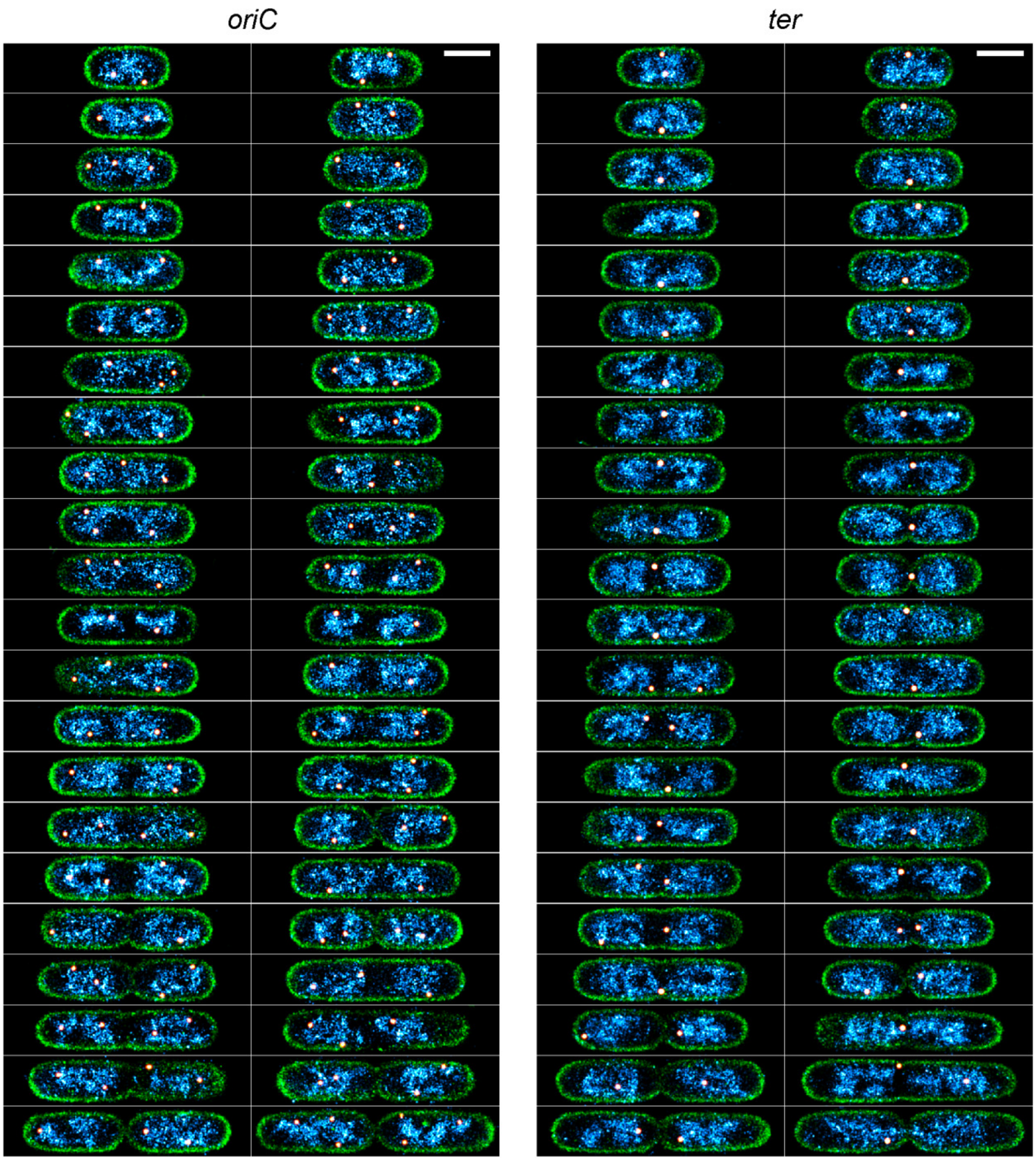

Figure S12: Spatial distribution of GFP tagged genetic loci in *E.coli* using a *parS*-ParB tagging system. **a,** *E. coli* MG1655 strains carrying a *parS/*GFP-Par tagged origin of replication (left side) or terminal region (right side) were sorted by size. Precisely localised genetic loci are false-coloured in red. Nucleoids were visualised using JF_{646}-Hoechst (cyan hot), while Nile Red was used to resolve the membrane (green) (scale bars: 1 µm).

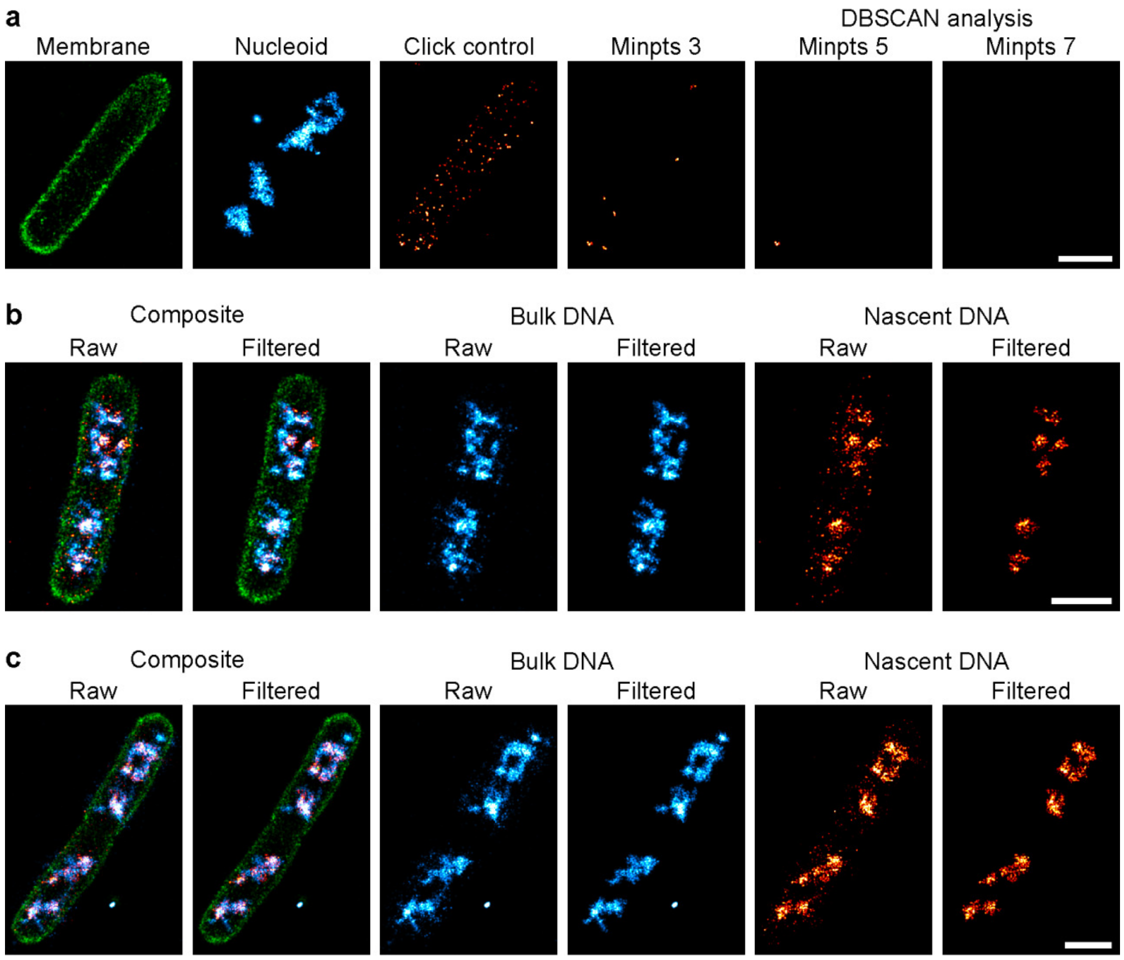

Figure S13: Filtering of low-density regions in nucleoid PAINT images using DBSCAN. **a,** Suitable DBSCAN parameters were determined using cells that were not treated with EdU, but clicked with Alexa Fluor 647 azide using the click-labelling protocol (see methods section). Imaging and fitting of Alexa Fluor 647 localisations (red) was performed similar to EdU-treated cells. The bulk chromosome was imaged using JF_{503}-Hoechst (cyan hot), while the membrane was resolved using Nile Red (green). ε was kept at 50 nm and the minimal number of localisations defining a clustered localisation (minpts) was adjusted until no clusters were detected in the *d*STORM image (minpts = 7). For bulk chromosome image, a value of minpts = 20 was determined to be suitable. These settings were used to process all pulse labelling data. **b,** Representative cell for a 2 minute EdU pulse. The low density regions are filtered efficiently without influencing the DNA structure. **c,** The determined parameters are also suitable to filter images of 5 min EdU pulse (scale bars: 1 µm).

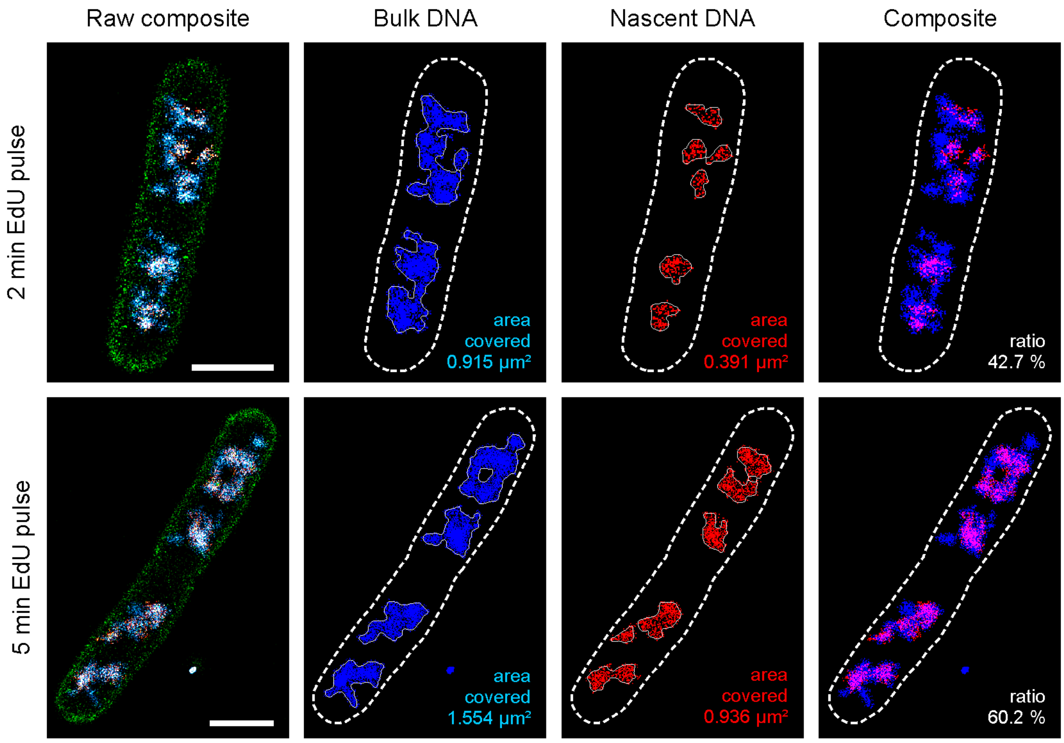

Figure S14: Coverage analysis of pulse-labelled and bulk DNA in multiplexed imaging experiments shown in Fig. 3. The area covered by bulk (cyan/blue) and nascent DNA (red) were determined in DBSCAN filtered images using the thresholding procedure described in the methods section. Determined outlines are shown as solid white lines, while dashed lines illustrate the cell boundaries. The analysis procedure robustly determines the distribution of nascent and bulk DNA for different pulse lengths. Longer EdU pulses hereby result in a higher coverage (scale bars: 1 µm).

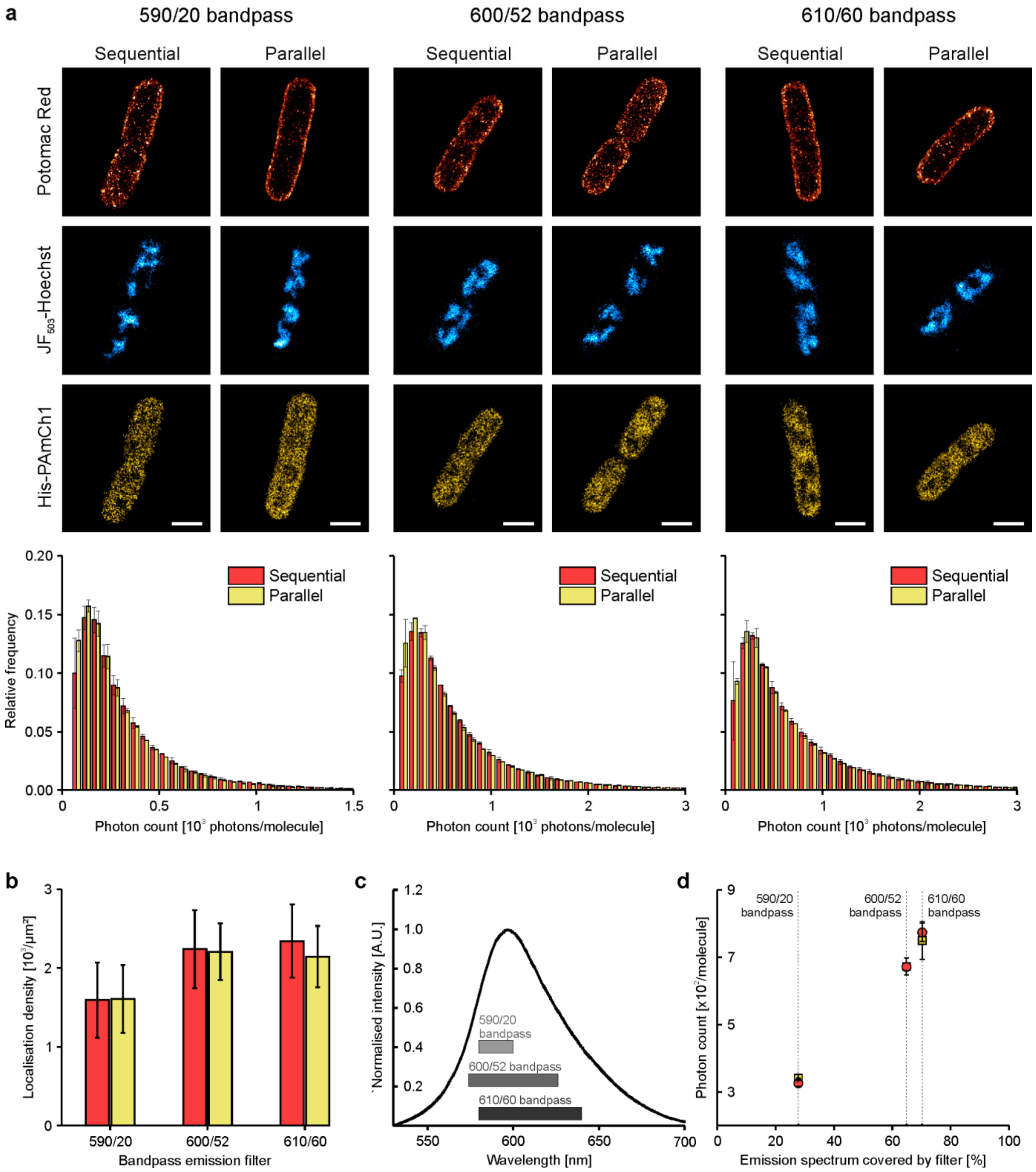

Figure S15: 3-colour SMLM imaging of cytosolic his-tagged PAmCh1, the bacterial membrane and nucleoid. The membrane was visualised using Potomac Red while JF_{503}-Hoechst was used to probe chromosomal DNA. During the sequential imaging, the PALM channel was recorded in absence of the PAINT labels. PAINT imaging was performed after buffer replacement and refinding of the PALM-imaged regions of interest. During parallel imaging, the PAINT labels Potomac Red and JF_{503}-Hoechst (400 pM each in 150 mM tris pH 8.0) were present in the imaging buffer also during PALM imaging. The three channels were recorded one after another without sample removal. **a,** Representative cells for sequential/parallel 3-colour SMLM imaging. 3 commonly used emission filters for PAmCh1 detection were tested for suitability and potential bleedthrough, which might occur in presence of the PAINT labels. For the filters tested, no bleedthrough could be detected visually (see **Fig. S16**). Furthermore, the intensity distributions of single PAmCh1 signals were found to be identical (bar

plots). Distributions show the average of two independent measurements (35,000 - 90,000 molecules per measurement) and error bars represent the standard deviation. **b**, Influence of PAINT labels on the number of detected PAmCh1 molecules. Due to the variance in cell size, the PAmCh1 localisation density [1/µm²] was determined for single bacteria both using the sequential (light red bars) and parallel imaging scheme (light yellow bars). Also here, no influence of the PAINT labels could be detected for all filters tested. Bars show the mean value and error bars the respective standard deviation (2 independent measurements, >15 bacteria per condition). **c**, Fluorescence emission spectrum of PAmCh1. The spectral range covered by the different bandpass filters is indicated by the grey bars. **d**, Intensity of single PAmCh1 signals in dependence of the different bandpass emission filter. The mean number of photons was calculated from the distributions shown in **a** and plotted against the relative integrated area of the emission spectrum covered by the respective filter. A clear linear correlation can be observed, indicating the robustness of our imaging and analysis parameters (scale bar: 1 µm).

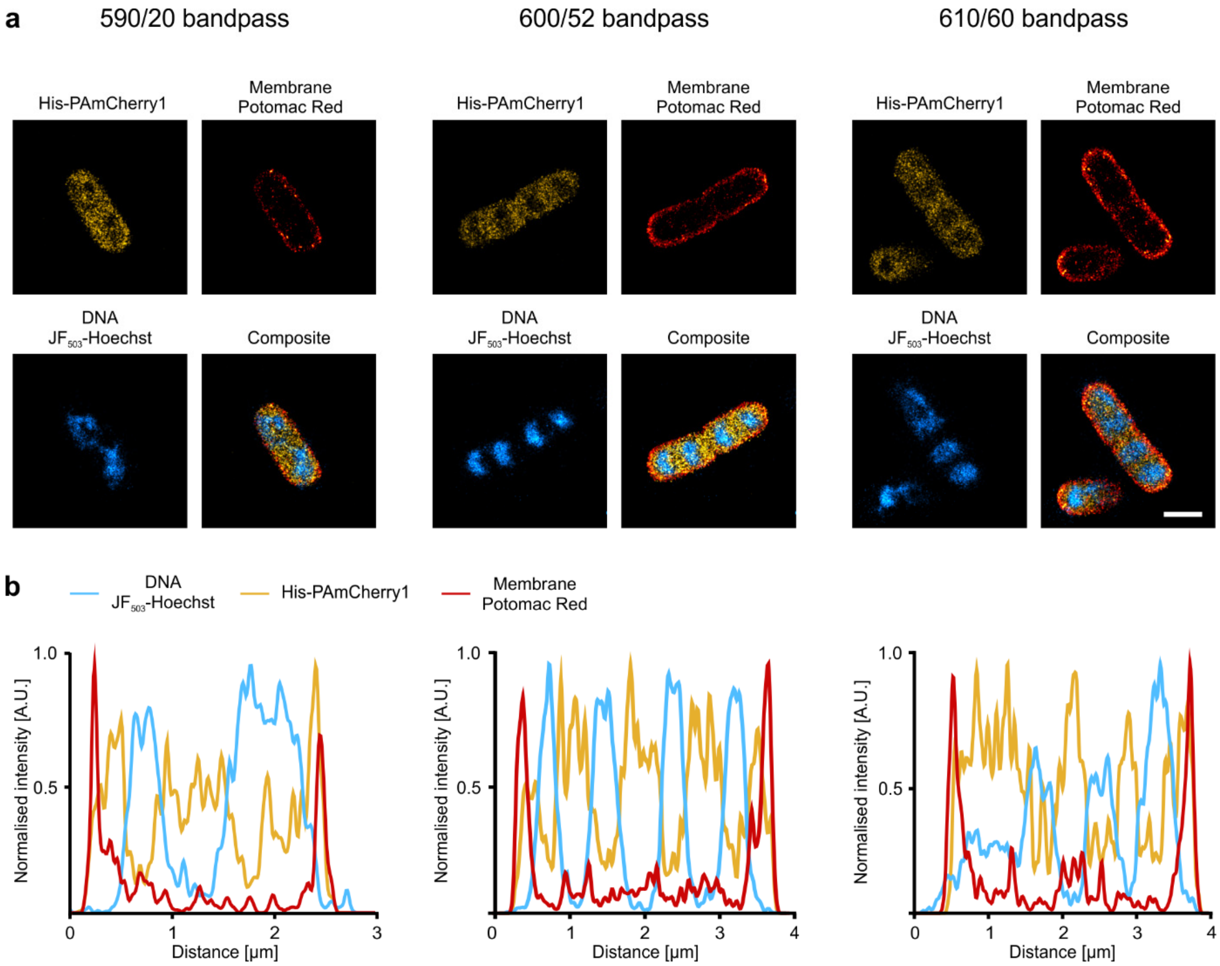

Figure S16: Visual inspection of crosstalk in parallel 3-colour SMLM imaging. **a**, Cytosolically expressed his-PAmCherry1 was imaged with PALM, followed by visualisation of DNA and the membrane using JF_{503}-Hoechst and Potomac Red, respectively. The presented images are chosen from the dataset contributing to **Fig. S15** (scale bar: 1 µm). **b**, Normalised intensity profiles of the bacteria shown in **a**. Profiles were determined along the bacterial long axis. The normalised intensity of the DNA is shown in blue, the membrane in red and PAmCherry1 in yellow. The distribution of the target structures recorded in different imaging channels are spatially exclusive, as shown both by the representative images in **a** and the respective profiles in **b**. This supports that there is no apparent crosstalk between the different imaging channels.

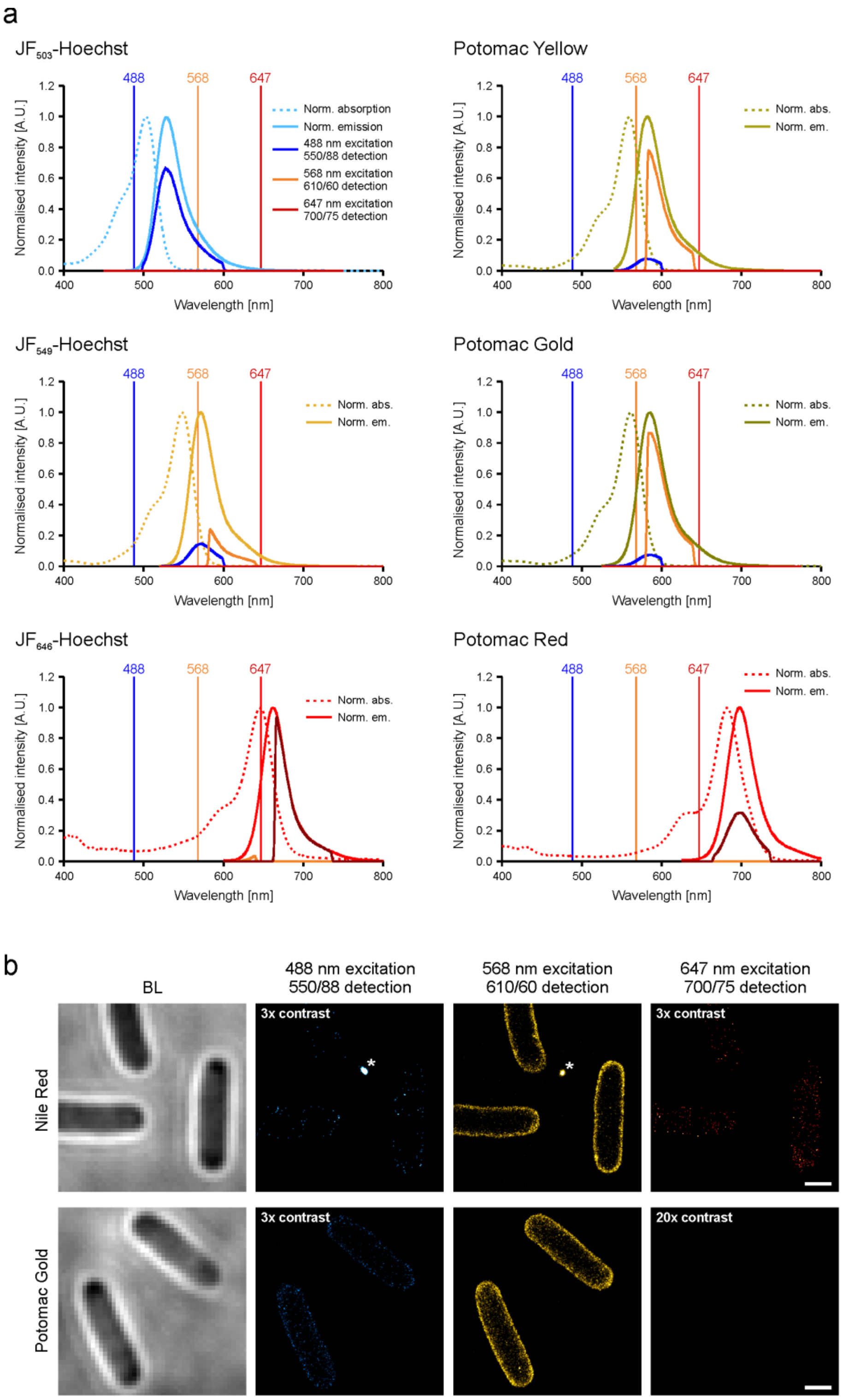

Figure S17: Theoretical investigation of crosstalk between the different imaging channels**. a**, Fractions of normalised fluorescence emission detected in the different imaging channels for the presented PAINT labels. The normalised absorption (dashed line) and emission spectra (dashed line) were used for calculation of the relative emission in each channel. Hence, the reference emission spectra were multiplied with the value for the normalized absorption at the respective excitation wavelength (488, 568 and 647 nm, indicated by coloured vertical lines) and the transmission of the filter used for signal detection (see **Table S2**). While no crosstalk is expected for JF_{503}-Hoechst, JF_{549}-Hoechst shows significant crosstalk with the blue channel and JF_{646}-Hoechst negligible crosstalk with the orange

channel. For the membrane PAINT probes, crosstalk with the blue image channel is expected for Potomac Yellow and Gold, while Potomac Red emits solely in the red channel. The integrated emission for the reference and calculated emission spectra were determined using the software Origin2018 (OriginLab, USA). Relative values for the detected emission in each image channel are provided in **Table S3**. **b**, Experimental investigation of crosstalk using the membrane PAINT dyes Nile Red and Potomac Gold. Because of the solvatochromic character of Nile Red, the crosstalk with other imaging channels could not be calculated as in **a**. To investigate potential crosstalk qualitatively, Nile Red or Potomac Gold in PBS were added to the sample and image sequences were recorded in the three channels by applying the respective illumination, acquisition and analysis settings used for parallel 3-colour SMLM imaging (**Fig. 5**, **Fig. S15**, **Fig. S16**). Only minor crosstalk of Nile Red can be observed in the blue and red imaging channels. The asterisk indicates a fiducial marker. For Potomac Gold, larger crosstalk is present in the blue imaging channel while no crosstalk in the red imaging channel. This is in very good agreement with the theoretical assessment shown in **a**. The contrast was increased for visualization, values represent the increase relative to the orange channel (scale bar: 1 µm).

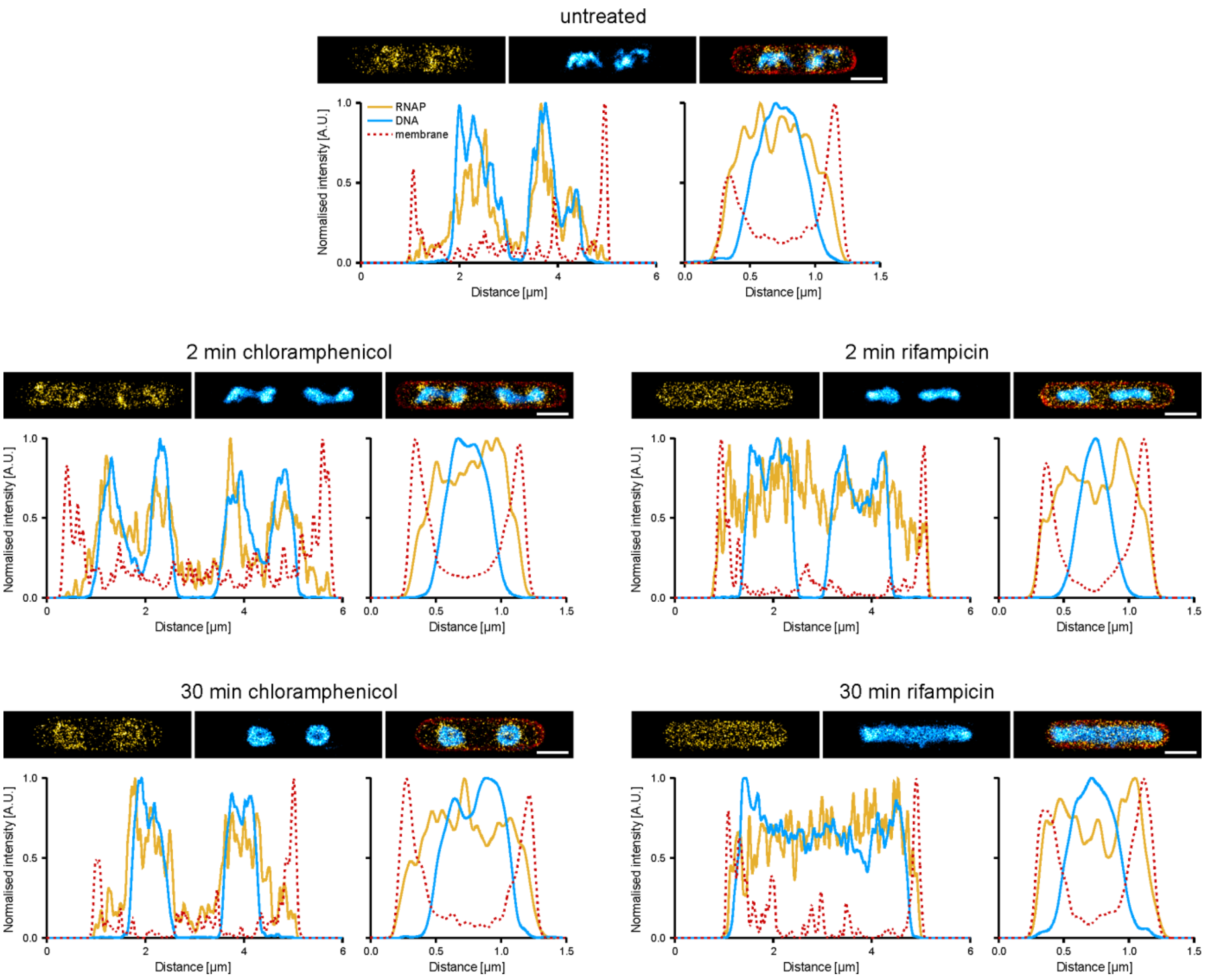

Figure S18: Normalised intensity profiles of representative, drug-treated *E. coli* cells. Cells were imaged for RNAP (PAmCherry1, yellow), the nucleoid (JF_{503}-Hoechst, cyan) and membrane (Potomac Gold, red) in parallel SMLM imaging mode. Intensity profiles were measured both along the bacterial long axis (left plot) and short axis (right plot). In untreated and chloramphenicol treated cells, RNAP molecules colocalize with the nucleoid as revealed by the SMLM images and long axis plots. The broader distribution of the RNAP signal compared to the nucleoid signal in short axis plots indicates that RNAP molecules transcribe active genes on the nucleoid surface, as proposed by several studies[6,7]. This association is maintained during the collapse of the nucleoid along the short axis (2 min) and the transition to a toroidal nucleoid (30 min). Inhibition of transcription initiation using rifampicin initially leads to a similar collapse of the nucleoid along the short axis (2 min) as for chloramphenicol treatment. Within the same time interval, RNAP detaches from the nucleoid and is distributed rather homogeneously throughout the entire cytosol (see line profile, 2 min). Longer rifampicin treatment (30 min) result in expanded nucleoids which populate the bulk cytosol in contrast to the condensed nucleoids observed in chloramphenicol treated cells (scale bars: 1 µm).

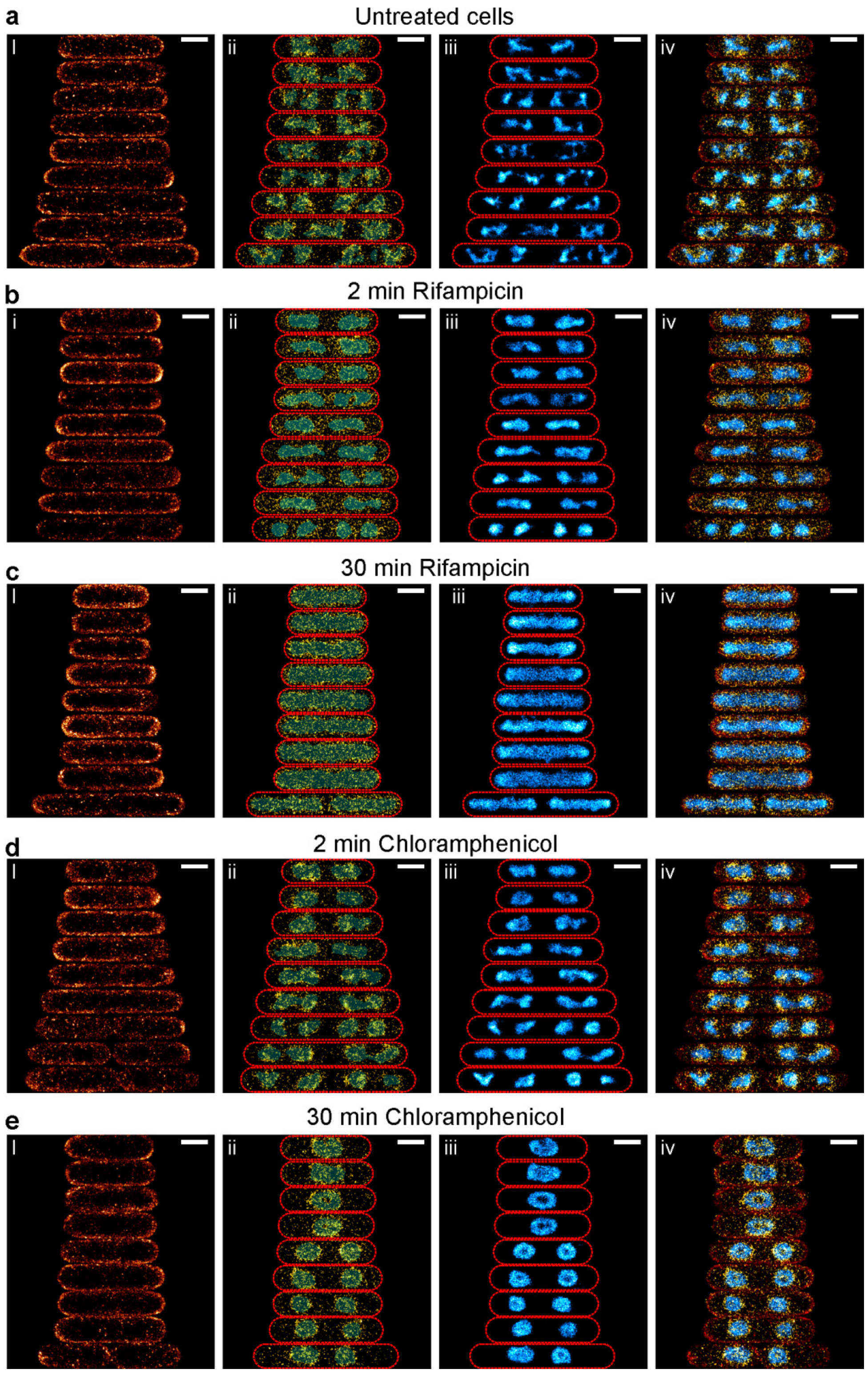

Figure S19: Parallel 3-colour SMLM measurements combining PALM and PAINT. Strain KF26 was grown in LB medium at 32°C, chemically fixed and imaged as described in the Methods section. Shown are membrane PAINT images (i, red), the PALM image of the RNA polymerase β' subunit (PAmCh1 fusion) (ii, yellow hot) and the PAINT image of the bacterial nucleoid (iii, cyan hot). The composite of the three SMLM channels is shown in iv. Red dashed lines indicate the membrane boundaries, estimated from the membrane PAINT image. The DNA PAINT signal is overlaid with the PALM signal (ii, transparent cyan) for better visualization of the RNAP positions relative to the

nucleoid. **a**, KF26 cells grown in absence of antibiotic. **b, c**, KF26 cells treated with 100 μg/ml rifampicin for the indicated duration. **d, e**, KF26 cells treated with 50 μg/ml chloramphenicol for the indicated duration (scale bars: 1 μm).

Table S1: Experimental localisation precision (determined using a nearest neighbour algorithm (NeNA), see Methods) of the DNA and membrane PAINT probes in different imaging buffers. The value for Alexa Fluor 647 was determined using pulse-labelled cells (5min EdU, **Fig. 3**). Due to the need for photoswitching, NeNA analysis for Alexa Fluor 647 could only be performed in the reducing buffer condition (tris/MEA). Values represent mean values and errors the respective standard deviation, calculated from multiple regions of interest in 3 independent measurements.

Target structure	PAINT dye	NeNA value [nm]	
		PBS	tris/MEA
DNA	JF_{503}-Hoechst	18.0 ± 1.0	11.9 ± 0.3
	JF_{549}-Hoechst	11.7 ± 0.4	9.6 ± 0.5
	JF_{646}-Hoechst	10.8 ± 0.7	11.7 ± 0.6
	Alexa Fluor 647	NA	10.8 ± 1.2
Membrane	Potomac Red	10.7 ± 0.4	11.1 ± 1.2
	Potomac Yellow	11.9 ± 0.8	10.6 ± 0.8
	5-carboxy-Potomac yellow	11.8 ± 1.1	11.1 ± 0.9
	Potomac Gold	12.3 ± 0.8	10.8 ± 0.9
	Nile Red	15.8 ± 1.7	17.0 ± 3.0

Table S2: Filter sets used for SMLM imaging of the different labels used in this study

Fluorescent dye/protein	Excitation filter	Dichroic mirror	Emission filter
GFP, JF_{503}-Hoechst	Z488/10 (Chroma)	HC Quad 410/504/582/669 (Semrock)	550/88 BrightLine HC (Semrock)
NR, Potomac Gold/Yellow, JF_{549}-Hoechst	Z488/568/647RPC (Chroma)	HC Quad 410/504/582/669 (Semrock)	610/60 ET Bandpass (Chroma)
PAmCh1	Z488/568/647RPC (Chroma)	HC Quad 410/504/582/669 (Semrock)	590/20 BrightLine HC (Semrock)<br>600/52 BrightLine HC (Semrock)<br>610/60 ET Bandpass (Chroma)
Alex Fluor 647, JF_{646}, JF_{646}-Hoechst, Potomac Gold	Z488/568/647RPC (Chroma)	HC Quad 410/504/582/669 (Semrock)	700/75 ET Bandpass (Chroma)

All components were purchased from AHF Analysetechnik (Germany).

Table S3: Calculated relative integrated fluorescence emission of the different DNA- and membrane-targeted PAINT labels in different imaging channels. Values were obtained by integrating the emission spectra shown in **Fig. S17**. NA = Not available.

**PAINT dye**	**relative fluorescence emission in % of the normalized emission spectrum**		
	**488 nm excitation 550/88 BrightLine HC**	**568 nm excitation 610/60 ET Bandpass**	**647 nm excitation 700/75 ET Bandpass**
JF_{503}-Hoechst	63.78	0.04	5E-4
JF_{549}-Hoechst	11.50	12.68	9E-4
JF_{646}-Hoechst	NA	0.84	51.02
Potomac Yellow	5.26	45.92	1E-3
Potomac Gold	4.76	54.50	3E-3
Potomac Red	NA	0.02	27.63

References

1. Grimm, J. B. *et al.* A general method to improve fluorophores for live-cell and single-molecule microscopy. *Nat. Methods* **12,** 244-50, 3 p following 250 (2015).
2. Nakamura, A. *et al.* Hoechst tagging: a modular strategy to design synthetic fluorescent probes for live-cell nucleus imaging. *Chem. Commun. (Camb.)* **50,** 6149–6152 (2014).
3. Grimm, J. B. *et al.* A general method to fine-tune fluorophores for live-cell and in vivo imaging. *Nat. Methods* **14,** 987–994 (2017).
4. Grimm, J. B. & Lavis, L. D. Synthesis of rhodamines from fluoresceins using Pd-catalyzed C-N cross-coupling. *Org. Lett.* **13,** 6354–6357 (2011).
5. Woodroofe, C. C., Lim, M. H., Bu, W. & Lippard, S. J. Synthesis of isomerically pure carboxylate- and sulfonate-substituted xanthene fluorophores. *Tetrahedron* **61,** 3097–3105 (2005).
6. Spahn, C., Cella-Zannacchi, F., Endesfelder, U. & Heilemann, M. Correlative super-resolution imaging of RNA polymerase distribution and dynamics, bacterial membrane and chromosomal structure in Escherichia coli. *Methods and applications in fluorescence* **3,** 14005 (2015).
7. Stracy, M. *et al.* Live-cell superresolution microscopy reveals the organization of RNA polymerase in the bacterial nucleoid. *Proceedings of the National Academy of Sciences of the United States of America* **112,** E4390-9 (2015).
